# Ultrawideband Antennas: Growth and Evolution

**DOI:** 10.3390/mi13010060

**Published:** 2021-12-30

**Authors:** Om Prakash Kumar, Pramod Kumar, Tanweer Ali, Pradeep Kumar, Shweta Vincent

**Affiliations:** 1 Department of Electronics and Communication Engineering, Manipal Institute of Technology, Manipal Academy of Higher Education, Manipal 576104, India; omprakash.kumar@manipal.edu; 2 Discipline of Electrical, Electronic and Computer Engineering, University of KwaZulu-Natal, Durban 4041, South Africa; 3 Department of Mechatronics Engineering, Manipal Institute of Technology, Manipal Academy of Higher Education, Manipal 576104, India

**Keywords:** ultrawideband (UWB), ultrawideband (UWB) notch antenna, frequency reconfigurable ultrawideband (UWB) notch antenna

## Abstract

Narrowband antennas fail to radiate short pulses of nano- or picosecond length over the broader band of frequencies. Therefore, Ultrawideband (UWB) technology has gained momentum over the past couple of years as it utilizes a wide range of frequencies, typically between 3.1–10.6 GHz. UWB antennas have been utilized for various applications such as ground-penetrating radars, disaster management through detection of unexploded mines, medical diagnostics, and commercial applications ranging from USB dongles to detection of cracks in highways and bridges. In the first section of the manuscript, UWB technology is detailed with its importance for future wireless communications systems. In the next section various types of UWB antennas and their design methodology are reviewed, and their important characteristics are highlighted. In section four the concept of a UWB notch antenna is presented. Here various methods to obtain the notch, such as slots, parasitic resonators, metamaterials, and filters are discussed in detail. In addition, various types of important notch antenna design with their technical specifications, advantages, and disadvantages are presented. Finally, the need of reconfigurable UWB notch antennas is discussed in the next section. Here various insight to the design of frequency reconfigurable notch antennas is discussed and presented. Overall, this article aims to showcase the beginnings of UWB technology, the reason for the emergence of notching in specific frequency bands, and ultimately the need for reconfiguring UWB antennas along with their usage.

## 1. Introduction

The electromagnetic spectrum has a finite range of frequencies that have been used for various forms of communication. The ultrawideband (UWB) has expanded this spectrum of frequencies for a broader range of applications by typically using nanosecond or picosecond pulses, which operate over a wide range of frequencies. Broadband antennas are incapable of passing such pulses for a short time period, leading to dispersion and distorted communication. Therefore, UWB technology has gained momentum over the past couple of years. The Federal Communications Commission (FCC) [1] defines a UWB spectrum as having a fractional bandwidth of typically greater than 0.2, where fractional bandwidth is defined by Equation (1).

(1) $$\mathrm{Fractional}\;\mathrm{Bandwidth}=2\times \frac{\left(f_{H}-f_{L}\right)}{\left(f_{H}+f_{L}\right)}$$

where the upper 10 dB cut-off frequency is denoted by $f_{H}$ and the lower 10 dB cut-off frequency is denoted by $f_{L}$.

An alternate definition of UWB technology requires the waveforms to have an absolute bandwidth of greater than 500 MHz. The pulses used in UWB technology have a short pulse width; they occupy a large bandwidth during transmission. UWB technology is typically used for video streaming, performing movie transfers, camera downloads, printing files on printers, and MP3 file transfers. However, the range of UWB pulses is limited from a few meters to a maximum of 50 m. Their speed ranges from typically 100 Kbps used in wireless sensor networks (WSN) to other higher speed communications typically of 1 Gbps.

The UWB technology has created a boom in the field of communication by proving its vitality in several applications viz., ground penetrating radar (GPR) for emergency communication during earthquakes or avalanches, detection of unexploded mines, health check of civil engineering structures such as highways and buildings, body area networks (BAN) and detection of water in subsurface soil layers for effective agricultural practices.

This article is arranged as follows. Section 2 describes the inception of applications that use the UWB technology for communication. Section 3 elaborates on the different designs of UWB antennas from 2000 to date. Section 4 presents an exhaustive survey of UWB notched antenna in order to mitigate the electromagnetic interferences (EMI) between the sub-bands. Since their inception, Section 5 presents the concept of reconfigurable UWB antennas and showcases the latest state-of-the-art antenna architectures.

## 2. UWB Antenna Technology

In [2], the authors have described the design of a bistatic synthetic aperture radar system for communication overboard a satellite. They used two antennae, one for transmission and the other for the reception of data with typical values of gain between 10 dB to 20 dB. The BISSAT (BIstatic Sar SATellite) antenna proposed was designed to work in the C-band. In [3], the authors outlined the issues that were faced for the usage of global positioning systems (GPS) aboard satellites. One of the primary concerns which ultimately paved the way for the need for UWB communication was the need to provide resilience to radio interference, i.e., for example, the International Telecommunication Union (ITU) had already designated the 2025 MHz–2110 MHz for Earth to space communication, and this frequency range interfered with terrestrial radio communication bands. This issue was therefore paving the way for UWB communication. In [4] the author described one of the preliminary usages of UWB communication which was for the detection of targets at sea. Their proposed technique utilized the characteristic of a large bandwidth of UWB antennas to remove the sea clutter and improve the target to clutter ratio for effectively detecting a target. In [5] authors also presented the potential of the usage of UWB technology for the future to enable ad hoc ubiquitous communication. The authors in [6,7] showcased a study conducted by Wireless World Research Forum (WWRF) on the need for new wireless interfaces and issues related to exhaustion of the then spectrum of 3G communication. This was a leading piece of research for the advent of UWB communications. 

The usage of UWB technology for car antennae used for navigation and other forms of communication was demonstrated by [8]. Both the antennae used for mobile and navigation communication were spiral in structure which operated at 350 MHz and 800 MHz, respectively. A significant application of the UWB technology was presented in [9] in the form of ground-penetrating radar (GPR). The authors of the article used a dielectric rod antenna that operated between the 1 GHz to 6 GHz frequency range, resulting in weak antenna–ground interaction. This enabled the novel design to be used for GPR applications. On similar lines in [10], the authors designed a Vee dipole antenna with resistive loads for UWB GPR applications. The wide range of frequency operations ranged from 500 MHz–8 GHz.

In [11] author presents a revolutionary article that proved the ability of UWB communication systems to coexist with Fixed Wireless Access (FWA) indoor systems operating in the frequency range of 3.5 GHz to 5 GHz. In [12], the authors investigated the effects of UWB technology on the existing 3G Wi-Fi networks. Their study showed through statistical analysis that if the UWB technology were not rolled out properly, the Wi-Fi throughput would be negatively impacted by 20%.

In the article [13], the authors have designed an adaptable ultrawideband wearable antenna for WBAN applications. The antenna design comprises a textile jeans substrate with a copper tape for the radiator patch and the ground plane. The proposed antenna showcases an optimum S11 value and radiation pattern under various bending conditions. The efficiency of the antenna in terms of return loss has been tested for planar and bending configurations. The antenna has an operating bandwidth between 2.2 GHz to 17 GHz for planar configuration. For bending configuration, the antenna radiated between 1 GHz to 1.5 GHz. For both configurations, there is a variation in the antenna twist states based on the direction of the twist. The radiation pattern is more stable along the Y-axis bending as opposed to the X-axis bending. In the article [14], the authors have designed a monopole antenna to diagnose a specific form of skin cancer using radiography. The proposed antenna has a dimension of (36 × 48 × 6.12) mm with an impedance bandwidth (8.2–13) GHz. The gain of the antenna is 7.04 dBi. The antenna has performed well in both planar and bending configurations. For the antenna with AMC, the radiation rate at the rear is lower when compared to the independent antenna. Further, the same has been noticed when the AMC antenna is placed on the body. Due to the body’s interference and absorption of energy, the AMC antenna’s gain when mounted on the body is lower when compared to the AMC antenna in free space.

In the article [15], the authors have presented the design of a flexible UWB antenna that showcases a bandwidth of 3.7 GHz to 10.3 GHz. With dimensions of 80 mm × 67 mm, the proposed antenna has a microstrip patch with two arc-shaped patches. This forms the entire radiator. Suppression of antenna loading due to the underlying body tissues has been achieved by using a full ground plane at the bottom of the antenna. The antenna performance has been studied in free space as well as human body environments. A VSWR of below 2 has been achieved in both environments. The VSWR remains constant even in case of bending of the antenna. Peak gain of around 4.53 dBi has been achieved with an overall efficiency of < 60%. The SAR also conforms to the requirement of lower than 2 W/kg with values of 0.147, 0.174, and 0.09 at 5 GHz, 7 GHz, and 9 GHz, respectively. However, slight discrepancies have been noted in simulated and actual measured parameters due to faulty fabrication. In the article [16], the authors have designed a coplanar waveguide (CPW) fed flexible UWB antenna which uses Mu-negative metamaterial, which can be used for wearable applications. They have designed an UWB antenna with 50 mm × 45 mm dimensions by fabricating it on two materials, namely, flexible FR4 and semi-flexible Rogers RT/duroid 5880. A bandwidth of 7.2 GHz to 9.2 GHz has been achieved. The overall gain of the antenna has been improved to 8 dBi by designing a 3 × 3 array. SAR values of 0.3 W/kg and 0.1 W/kg have been achieved for 3 GHz and 10 GHz, respectively.

Authors in [17] have presented the design of a textile antenna for wearable medical imaging applications. The antenna has been designed in a triangular shape with parallel slots to enhance radiation in the UWB range of frequencies. Flexible polyester has been used for constructing the antennas. In [18], the authors presented yet another novel application of UWB antennas for Wireless Body Area Networks (WBAN). The antennas designed proved their usage owing to their little thickness of around 0.5 mm. The authors proposed two physical designs of antennas, viz., a coplanar UWB disc monopole and UWB annular slot antennas. The antennas operate in the 3.11–10.6 GHz frequency range. In [19] authors also investigated the usage of UWB technology for WBAN applications. They developed a novel Swastika slot-ultra-wideband (SS-UWB) patch antenna, which operated in the frequency range of 3.1–10.6 GHz. Also presented the design of a high-fidelity UWB antenna for WBAN applications. They implemented a structure with two layers of patches with a 2 mm thick substrate to enable radio communication over a range of 1 m. In [20], the authors present yet another flexible UWB antenna used for BAN. It is created by integrating a conductive tissue-like fabric on polydimethylsiloxane (PDMS) substrate. The antenna operates at a frequency range of 2.0–2.5 GHz with an efficiency of 70%. Authors in [21] designed a UWB of 4.68 mm thickness to operate in the frequency range of 4.33 GHz to13 GHz. The authors have reported that the specific absorption rate (SAR) values of the antenna reduce by 98.3% when metamaterial is used as the substrate. 

Hossain et al. in [22] have designed a bird face monopole ultrawideband antenna. It showcases an operating bandwidth of 3.10–12.30 GHz. The antenna gain variations are not uniform. The cross-polarization levels are not discussed. In [23] authors proposed the usage of antenna arrays aka vector antennas to increase the bandwidth of UWB communication systems. They designed an antenna array comprising a loop antenna and two orthogonal bowtie antennas that worked in a 3.6–8.5 GHz frequency range. Moreover, UWB antennas have also been designed for USB dongle applications [24]. The antenna used is a U-shaped monopole antenna with a metal plate with a ground plane made of foam. The size of the antenna is relatively compact for the purpose of the dongle. The antenna design provides an ultra-wide bandwidth of around 7.6 GHz.

In [25], authors described a yet another revolutionary application of UWB technology: the designed sensing through the wall systems. This proved to be an integral technology for military applications in urban terrain. The authors examined the feasibility of UWB signals through wall imaging in [26]. The authors used UWB noise waveforms as a probing signal with the principle of heterodyne correlation to image objects behind a wall successfully. Authors in [27] employed the short-term Fourier transform (STFT) of UWB frequencies to sense targets across foliage. Similarly, in [28,29], the authors provided an insight on the usage of support vector machine (SVM) in integration with UWB signals and Singular value decomposition (SVD) algorithm, respectively, to classify human presence through the foliage.

Authors in [30,31] investigated the usage of UWB technology for mobile terminals. The former designed a stubby antenna that resonated in a band of 1.0–6.0 GHz, and the latter provided a survey of reconfigurable adaptive CMOS circuits using UWB signals for 4G mobile applications. The usage of UWB sensor networks for target localization in the mining industry was investigated in [32].

As the UWB technology began carving its niche in the wireless domain, [33] presented a study of its interference with WiMAX systems typically in 2.5–3.5 GHz frequencies. They concluded that UWB signals with an Effective Isotropic Radiated Power (EIRP) of 83 dBm/MHz or less for a distance of 1 m would not pose any interference to WiMAX signals. Authors in [34] showcased the viability of integrating Bluetooth with UWB technology over the MAC layer and PHY layer of transmission of signals. 

The indoor and location tracking applications in harsh environments using UWB technology were investigated by [35,36], respectively. In [35], authors stated that for mission critical scenarios such as rescue requiring drastic measures for mission planning and execution, it was essential to have systems that could operate in a wide range of frequencies for which the UWB technology was most suited. In [36] the author extended to present the feasibility of UWB signals for tracking critical resources in construction environments. It is used as a support tool for managing construction sites. 

In [37,38,39,40], the authors proved the feasibility and significance of UWB signals to penetrate through foliage/debris in a critical situation such as an earthquake or a dilapidated coal mine and detect the presence of life in humans. The fundamental advantage these researchers tapped in was the excellent penetration capability of UWB signals.

UWB technology was also proposed to be used in the medical field for Microwave Radiology Imaging (MRI) application in [41]. The authors presented a Slotted Triangular Flared (STF) patch mounted on a substrate of Teflon. An in-phase reflector increased the bandwidth of the system, causing it to work in the UWB range of frequencies. In [42], the authors have revolutionized the medical realm by designing a UWB antenna with slots for breast cancer detection. The advantage of using UWB for microwave imaging is that it provides non-ionizing signals with large bandwidth. A mini rectangular patch antenna has been used for this purpose.

UWB technology has also been proposed for detecting voids in specimens of concrete [43]. This has been achieved by designing a Vivaldi antenna to operate within a frequency range of 1–30 GHz. The designed antenna has obtained high resolution images.

The authors in [44] showcased the feasibility of UWB communication systems to become an integral part of 5G wireless systems. A rake receiver for indoor applications has been designed to mitigate multipath effects and provide throughputs.

UWB technology has also found its application in Near Field imaging applications used in Synthetic Aperture Radar (SAR) [45]. The authors of this article have designed an antenna with two dipoles in the orthogonal configuration with elliptical arms and a modified configuration for feeding. The antenna has been used for the application of monitoring perforations in an oil well wall. In [46] authors have examined the feasibility of living in smart homes integrating UWB technology with deep learning for the elderly. Recognition of activities performed by seniors is done using three UWB radars along with a deep learning model and a voting system. Table 1 describes the emergence of UWB technology and its applications in Section 2 above.

## 3. Progress/Emergence of Design of UWB Antenna

The need for UWB technology was born with the idea of a system encompassing a wide bandwidth of operation and very low interference with existing technologies such as Wi-Fi, WiMAX, and GPS. Section 1 described the varied applications developed over the years using UWB antennas due to their superior bandwidth, gain, and low interference features as opposed to other wireless technologies. Table 2 lists the important UWB antennas proposed in the literature with their design specification, advantage and disadvantages. 

In [47], the authors have conducted an extensive survey on the promises offered by the UWB radio technology (UWB-RT), the primary one being to reduce the problem of a narrow spectrum. The authors have proposed to harness the ability of UWB technology to work in conjunction with other 3G wireless technologies.

In [48], the author designed a UWB antenna to remove the scattering of waves using resistive sheets for a slot line bowtie hybrid (SBH) antenna. The authors have employed the Genetic algorithm (GA) to stabilize the antenna’s beamwidth. A lower radar cross-section (RCS) is obtained by exploiting the fact that resistive sheets are almost transparent of EM fields.

In [49], the authors have also been able to obtain a uniform radiation band over the entire bandwidth with a constant gain of 10 dB by designing a diplexer with two patch antennas stacked one over the other. In [50], authors have proposed a planar monopole antenna that is shorted and has a bevel. Their design demonstrates an increase in bandwidth by combining beveling along with shorting the monopole antenna. A bandwidth of 50 to 1 from 300–15,000 MHz is achieved by [51] by employing the genetic algorithm to generate the shape of the antenna and the impedance loads connected in series.

The authors in [52] have analyzed the usage of the finite-difference time-domain (FDTD) method to design horn-fed bowtie antennas (HFB) to work in the UWB frequency spectrum. A wide bandwidth has been achieved, making these antennas suitable for GPR applications. In [53], authors have investigated the need for slotting in the conventional UWB antennas. They utilized the planar inverted cone antenna (PICA), which is used in the UWB frequency range. The PICA antennas are modified by inserting two holes of the circular shape to change the flow of current on the metal disk, in turn improving the bandwidth of the radiation. In [54], authors have also investigated the usage of the FDTD technique to design two conical plane transmission lines. Their surge impedance has been calculated for various geometries and various dielectric loads. These antennas radiate in the UWB range and are suitable for GPR applications. A circular planar monopole with microstrip line feeding has been designed in [55,56]. Their antenna showcases a return loss of 10 dB with a stable radiation pattern. The impedance characteristic of an exponentially tapered UWB TEM horn antenna is improved using a balun of microstrip-type in [57]. The proposed design showcases a bandwidth three times more than the conventional linearly tapered TEM horn. In [58], the authors have designed a planar UWB antenna with an elliptical radiator feed using a microstrip line. Both the radiator and the feed line are mounted on the same ground plane. The authors of this article have been able to generate a bandwidth of 110.9% gain from 1.34 dBi to 5.43 dBi within an operating frequency range from 3–10.6 GHz. UWB operation is achieved using magnetic coupling of two sectorial loop antennas which are arranged symmetrically in [59]. After optimizing the design, a VSWR of around 2.2 across 8.5:1 frequency is achieved. In [60], a trident-shaped feed has achieved UWB characteristics with a bandwidth of about 10 GHz. This feed is integrated with a square monopole in the planar structure fabricated on a single metal plate.

**Table 2:** The emergence of design of UWB antenna.

Reference No.	Size (mm^2^)	Gain (dBi)	Efficiency	Advantage/Disadvantage	Reason for UWB Performance	Design Methodology	Operating Frequency Range (GHz)
[48]	-	-	-	Advantage: Good impedance matching and compact in size Disadvantage: Absorber treatment is required in E and H planes to achieve desired performance	Bowtie TEM horn	Genetic algorithm	3.1–10.6
[50]	141.4×141.4	-	-	Advantage: High bandwidth Stable radiation pattern Disadvantage: Large size	introduction of a bevel	Cutting slots in each side of the planar element	0.8–10.5
[51]	1220×1220	-	20–100	Advantage: Elliptical polarization and near hemispherical coverage Disadvantage: Low efficiency at lower frequencies	A genetic antenna consisting of a set of wires connected in series and with impedance loads	genetic antenna	0.3–15
[52]	2550×2550	-	-	Advantage: Important design characteristics can be measured using this FTDT model Disadvantage: Complex antenna	planar bowtie dipole with the feed point being raised off the ground	FDTD technique	3.1–10.6
[55]	71×86	12.5	-	Advantage: Good group delay Disadvantage: Unstable radiation pattern	The broadband microstrip line to the slot line transition causes a large impedance bandwidth.	Tapered slot	3.1–10.6
[56]	50×42	8	-	Advantage: Good frequency characteristics in the UWB range Disadvantage: Poor polarization	Circular disc monopole fed by microstrip line	Use of the feed gap, the width of the ground plane, and the size of the disc.	2.6–10.1
[57]	1000×3	11	-	Advantage: Improves bandwidth of TEM horn antenna Disadvantage: High cross-polarization	TEM horn antenna with exponential tapering with a balun of microstrip type	Use of linearly tapered and exponentially tapered structure	0.6–15.7
[59]	200×200	8	-	Advantage: Excellent polarization and good impedance bandwidth Disadvantage: Gain decreases from 6 GHz to 10 GHz	Magnetic coupling of symmetrical sectoral antennas of loop shape	Two Sectors	2–16
[60]	150×150	7	-	Advantage: Easily fabricated using the single metal plate and Cost-effective Disadvantage: Large size	Trident-Shaped Feeding Strip	Combination of square planar monopole antenna with a trident-shaped feeding strip	1.4–11.4
[61]	1000	8	-	Advantage: It doesn’t need an anechoic chamber for measurement Sensitivity to noise is low Disadvantage: Efficiency is low	Use of conducting wire antenna as a pair of scissors	scissors antenna consists of conducting wires	0.2–4
[62]	6.5×66.3	6	-	Advantage: Uniform radiation pattern and less distortion in the baseband signal Disadvantage: The tradeoff between antenna performance and miniaturization	The tapered-slot feed acts transform the impedance and directs the maximum radiation from the slot line to the radiating slot	Tapered slot	3.1–10.6
[63]	40×38	7	-	Advantage: Omnidirectional radiation pattern Disadvantage: The time-domain analysis is not performed	CPW fed with a U-shaped tuning stub and elliptical slot	Elliptical slot with U shaped stub	3.1–10.6
[64]	136.2×66.4	12	-	Advantage: High gain Disadvantage: Large size	Double exponential tapered slot on LCP gives end-fire radiation in UWB range of frequency	Double exponential tapered slot	3.1–10.6
[65]	126×60	-	-	Advantage: Stable radiation pattern up to 6 GHz Disadvantage: Pattern not stable at higher frequencies	planar rectangular monopole antenna with a sleeved transmission line with the displaced feed point	sleeved transmission line- fed rectangular planar disc monopole antenna with a small ground plane	0.5–9.0
[66]	30×11.5	2.2	-	Advantage: Suitable for mm-wave applications Disadvantage: Less gain	Resonators of varying lengths are added along the feedline.	Stepped slots	6–22
[67]	120.3×24.8	7	80	Advantage: Large bandwidth and low pulse distortion Disadvantage: Large size and complex structure	A tapered loop is formed from the CPW to CPS to increase the operating bandwidth	Circular tapered slot	3.1–10.6
[68]	62×52	6	-	Advantage: Impedance bandwidth of 144.8% and stable radiation pattern Disadvantage: Large size	A circular slot with a new moon-shaped strip connected to the feed line causes a wide bandwidth. This is created to stabilize the radiation pattern at higher frequencies.	Circular slot with moon-shaped strip	2.4–11
[69]	30×46	3.8	-	Advantage: High efficiency and good omnidirectional radiation pattern Disadvantage: $S_{11}>-10\;\mathrm{dB}$ for 3.1 to 4 GHz	Beveling the bottom border of planar monopole antenna	Slot cut in the ground plane	3.1–12
[70]	160×108	18	-	Advantage: Good gain and directivity. Negligible cross-polarization Disadvantage: Weak phase dispersivity	Slot etching on the ground plane with a central point feed	leaky slot line	4–40
[71]	50×46	1.54	-	Advantage: Simple structure and stable radiation pattern Disadvantage: Less gain and large size	The curves of the antenna edge by a binomial function which gives the wider impedance bandwidth	Use of a binomial function	2.5–10.9
[72]	251.32	7	-	Advantage: Constant high gain and good transient response Disadvantage: Complex design	Biconical dipole with metallic reflector	Tapered stripline balun	3–20
[73]	90×90	8.2	-	Advantage: Minimum ringing and dispersion of pulse in the time domain Disadvantage: Large dimension	Elliptical monopole antenna	Elliptical monopole antenna	12–12.6
[74]	47×45	9.9	-	Advantage: Good gain Disadvantage: The radiation pattern is moderate.	Suspended (Plate antenna) structure	It consists of four top plates connected to a bottom plate using four vertical strips.	3.1–4.8
[75]	100×100	5.2	-	Advantage: Simple design, cost-effective, and mechanically robust Disadvantage: Large size	Suspended plate antenna with the shorting wall together with the L-probe feed	Parasitic L-shaped plate	3–12
[76]	30×8	4	-	Advantage: Omnidirectional radiation patter with low cross-polarization Disadvantage: High insertion loss below 5 GHz and limited gain therein	The planar monopole has strong vertical currents, which leads to large impedance bandwidth. A triangular feeding strip is used.	Rectangular monopole with an equal-width ground plane	2.7–16.2
[77]	30×20	4.13	-	Advantage: Low VSWR, stable gain and linear phase Disadvantage: There is a reduction in radiation efficiency due to an increase in input impedance in the mid UWB band.	There are electric and magnetic radiators. Energy stored in matching stubs is radiated because of the CPW-slot line transition.	Two rectangular slots	3.1–10.6
[78]	71×71	4	-	Advantage: Simple feeding structure and good bandwidth Disadvantage: Large size	The polygonal slot results in bandwidth enhancement	Polygonal slot	1.8–5.9
[79]	24.5×24.5	5.9	-	Advantage: Small size with impedance bandwidth of 122% Disadvantage: The radiation pattern is quite unstable.	Asymmetrical beveled rectangular patch with a U slot causes enhancement in bandwidth	Asymmetrical rectangular patch with U shaped slot	2.9–12.1

Note: - indicates not applicable.

The Scissors antenna has been designed by [61] for transient UWB applications. It consists of conducting wires which radiate ultrashort pulses having low dispersion. It has been proven to have a better radiation characteristic in comparison to conventional harmonic measurements. A UWB within the frequency range of 0.5–9 GHz has been designed in [63]. The authors have used a monopole in the shape of a rectangular disc which is fed with a sleeved transmission line with a small ground plane to achieve this high bandwidth. A UWB usable for handset applications with a folded metallic element connected to a printed section has been proposed in [69]. Various paths for current have been created, thereby increasing the dimensions of the antenna. The antenna has a quasi-omnidirectional radiation pattern over the entire UWB frequency range of operation.

The UWB leaky lens antenna has been designed in [70], which utilizes the property of Cherenkov radiation occurrence in a slot that is printed in the conjunction of infinite homogeneous dielectrics. This results in a directive antenna that has non-dispersive characteristics.

The binomial function has been used to design the edge of the curve of the planar antenna designed in [71]. Different orders of the binomial function have been used along with varying widths of the gaps between the antenna and the ground plane. The proposed antenna showcases an omnidirectional pattern of radiation in the UWB range of frequencies. In [72], a rod antenna with a metal reflector that operates in the UWB range has been presented. The antenna is fed with a biconical dipole with a microstrip line balun. This design showcases a bandwidth of operation from 3–20 GHz. It also exhibits very less dispersive effects.

A planar UWB elliptical monopole antenna has been presented in [73]. An ellipticity ratio of 1.1 gives a UWB ratio of 12.4:1 as opposed to 10.2:1 as of a circular monopole antenna. A suspended plate antenna (SPA) has been designed in [74]. The bottom layer plate is suspended over the ground and has a feed that is tapered towards it. The upper layer has four radiation plates, each connected to the bottom plate using vertical metallic strips. The proposed antenna radiates in the frequency range of 2.8–6 GHz with a stable gain. A UWB plate antenna with a wall for shorting which is attached to the radiator has been presented in [75]. The antenna is excited with an L-shaped plate. Due to the electromagnetic coupling between the feed plate and the radiator, radiation over the UWB range of frequencies has been achieved. The rest of the antennas tabulated in Table 2 illustrate the various characteristics owing to which a UWB performance has been achieved. Table 2 tabulates the designs of simple UWB antennas along with their key design features and radiation characteristics.

As presented in this section, it may be observed that each of the antennas radiates in the UWB range of frequencies without notching in any range of frequencies. However, with the improvement in UWB technology, the need for notching in specific frequencies such as the Wi-Fi, WiMAX was noticed to avoid interference. This led to the emergence of creation of slots of different shapes and sizes in different positions to generate notches. To mitigate the effect of EMI between the other devices operating within the UWB frequency range, Section 4 describes in detail the various literature available for slotted antennas.

## 4. The Emergence of UWB Notch Antenna

UWB systems present the greatest challenge of overcoming interference with narrowband systems due to their wide bandwidth [80]. Existing technologies such as WiMAX, which operates in the range of 3.3–3.7 GHz, Wireless LAN, which operates in the range of 5.15–5.35 GHz, C and X bands of the ITU, which operate in the 7.25–7.75 GHz and 8.025–8.4 GHz, respectively, pose serious possibilities of strong interference to UWB systems. Strong interference due to narrowband systems can result in strong disturbances similar to background noise. This factor of noise increases the power spectral density of the signal, thereby reducing the capacity of the UWB system.

The interference between UWB devices and narrowband devices that hampers the communication systems’ overall performance is mitigated using some novel modifications on the antenna structures. Several techniques are proposed to design UWB antennas with frequency stop or frequency notch characteristics. Depending on the application and the nature and bandwidth of the rejection band, researchers developed novel antennas with an integrated filtering mechanism that provides the desired response. Several antenna designs are reported which provide frequency notch response. Some common techniques [81] used to obtain frequency notches are

- Modifications on the radiator.
- Modifications on the ground plane or signal line.
- Integrated filter techniques.
- Metamaterial-inspired resonators.

Modifications on the radiator and ground planes in the form of slots, stubs, and slits are the most common techniques to achieve frequency notches in a UWB-printed antenna. The literature proposes many configurations using monopole antennas of the planar type, which have a modified radiator characteristic. These configurations have been used as a common practice to alter the current path in printed antennas, which affects the antenna’s input impedance. The aforementioned different geometrical structures with varying sizes yielded different notching results by changing the current distribution on the radiator of a UWB antenna.

In [82], a CPW-fed rectangular antenna with a T-shape stub and rectangular slot is proposed. The proposed design gives a notch between 5–6 GHz. An ultrawideband notch antenna with a single parasitic strip is proposed in [83]. Notching is achieved by utilizing an Elliptic slot with three steps, attaching a parasitic strip to the lower end of the antenna, and varying its length. It shows a notched band between 5.1–5.8 GHz.

A monopole antenna with a C-shape slot on a patch in [84] causes a single notch in UWB operating band. Slotted radiating surfaces like T-shaped slot [85], octagonal slot [86], two arc-shaped slots [87], Tapered slot [88], and U-shaped slot [89] are used for rejecting undesired bands from the UWB spectrum. Different geometrical shapes and sizes with slots like fractal geometry in the hexagonal monopole with Y-shape slot [90], Koch Snowflake iterative design with Snowflake slot [91], Fractal Koch structure [92] with T-shaped stub, and Fractal slot [93], two elliptical patches on a hexagonal radiating patch with two C-shape slots [94] are utilized to reject the undesired frequency bands. A triple-band UWB notch antenna is proposed in [95] by using decoupling structure, multi-slit, and multi-slot concepts.

Modifications on the ground plane or signal line were also used to obtain notches in the operating frequency band of the UWB antennas. A UWB notch antenna using open-ended slot is proposed in [96]. A staircase shape monopole UWB notch antenna is proposed in [97]. Wide bandwidth and notching are achieved by utilizing a half-bowtie radiating element, a U-shaped staircase slot, and a modified ground. An antenna with one band-notch has been proposed in [98] and [99]. In [99], the antenna has an arc-edged patch with a partially modified ground plane. The microstrip-fed dipole of semi-elliptical shape antenna [100], CPW and ACS-fed monopole with DGS [101], CPW-fed L-slot microstrip antenna [102], offset microstrip-fed UWB antenna with L-shaped slits on the ground [103], and slots in the shape of arcs in monopoles [104] have been utilized to reject the undesired frequency bands. A three-band notch antenna has been proposed in [105]. In this design, two bevels in the patch, two bevels in the ground are used to get wide bandwidth. The usage of two round slots of half-wavelength along with two slots of C shape in the ground plane gives a notch at the desired frequency.

Several band rejection techniques like asymmetrical resonator in the feed line, parasitic strip on the radiator, inverted-L slot on the ground plane [106], tapered edge on the ground plane and rectangular slots on patch [107], Circular-stub-based multimode-resonator UWB filter [108], resonant parallel strip (RPS) [109], four stubs and stepped slot in the ground plane [110], H-slot patch for radiation and a slot of U shape for feeding [111], slot resonators on Y-shaped monopole radiator [112], parasitic resonator [113], meander line resonator with open ended stub loading [114], inverted U-shaped and I-shaped slots each of half guided wavelength on radiator [115], EBG structure [89,116] Notch filter in the patch [117], parasitic loading and slot techniques [118], DGS and orthogonal polarization [119], inverted L-shaped stub resonator [120], and a stub with an open end with a meandered resonator along with defective ground structure [121] are reported in the literature to receive band notch characteristics.

Recently, metamaterial (MTM)-inspired resonators have been utilized to achieve notching in UWB operating band. Due to the advancement in consumer electronics, the antenna on demand must have high channel capacity, gain, bandwidth, and compact size. Many techniques have been proposed to improve antennas’ performance over the last decade. One of such techniques is metamaterials. Due to their superior properties compared to natural materials, Metamaterials have achieved enormous attention from researchers [122].

MTM inspired antenna is designed using the unit cell structures into the ground plane or radiator. This unit cell structure acts as a resonant structure in the antenna [123]. A circular split-ring resonator (C-SRR), Square split-ring resonator (S-SRR), Hexagonal split-ring resonator (H-SRR), and Rotational circular split-ring resonator (RC-SRR) are the most common SRR configurations used for frequency notch applications. Many metamaterial-inspired resonators like C-SRR in the signal line [124], Complementary Split Ring Resonator slot [125], Symmetrical Split Ring Resonator [126], the combination of complementary split ring resonators (CSRR), split ring resonators (SRR) and DGS [127], SRR [128], open loop resonators [129], elliptic split ring resonator (ESRR), and a round split ring resonator (RSRR) along with U-shaped parasitic strips near the signal line [130] are reported in the literature to receive band notch characteristics.

Based on the above techniques, various metamaterial-based UWB notch antennas are designed [131,132,133,134,135,136,137,138,139]. In the paper [131], UWB antenna is designed using double-slotted ring resonators, circular (C-SRR) and square (S-SRR). It has impedance bandwidth from 3.5–9 GHz and a gain of 5 dBi. It shows multiple band frequencies rejection and stable radiation patterns. In the paper [132], a UWB monopole notch antenna is designed using SRR on the ground plane. It has an operating band from 3.1–10.6 GHz It has a notched band from 5–6 GHz and functions for WLAN. In [133], authors have proposed a dual-band UWB notch antenna using a CSRR on the patch and the SRR on the ground plane. It is useful for WiFi 6E and 5G communication. In [134], a UWB antenna is proposed using an annular SRR slot and a rectangular SRR slot to achieve dual notched band characteristics. In [135], a UWB notch antenna was designed for 5G, WLAN, and Satellite downlink bands applications. The authors use EBG structures to notch 5G and WLAN bands and 2 SRR to notch Satellite downlink bands. A triple-band planer antenna using metamaterials has been proposed in [136]. This design uses the annular defected ground to get wide bandwidth. The multi resonant metamaterial cell usage gives a triple band of 2.38–2.48, 3.37–3.79, and 4.36–6.06 GHz. A single-notch UWB MIMO antenna using a parasitic decoupler has been proposed in [137]. This design uses a parasitic decoupler to improve the isolation between antenna elements. The U-shape slot usage in the radiator gives a notched band of 4.2–5.8 GHz. A triple band-notched UWB antenna has been proposed in [138]. This design uses a SRR in the ground to notch WiMAX (3.3–3.8 GHz) and WLAN (5.15–5.825 GHz). The U-shape slot usage in the feed line gives a notched band of X-band (7.25–8.395 GHz). In [139], a UWB notch antenna was designed for rejection of the WLAN band. The authors use slotted complementary SRRs structures in a circular patch on a partial ground plane to notch the WLAN band of 5.5 GHz.

Table 3 tabulates the aforementioned sharp band rejection techniques and their key design features and radiation characteristics to avoid overlapping the UWB with the interference bands.

## 5. Frequency Reconfigurable UWB Notch Antenna

The previous two sections have showcased the evolution of UWB antennas and UWB notch antenna and their design methodology. With the emergence of reconfigurable circuits, UWB technology also adapted the concept of frequency reconfiguration and several frequency-reconfigurable UWB notch antennas have been designed.

### 5.1. Principle of Antenna Reconfiguration

Over the years, antennas can be divided into broad categories, which include wire antennas, waveguide antennas, log-periodic antennas, microstrip antennas, reflector antennas, and aperture antennas. According to the need of the design, these basic antenna structures are used to develop new structures to suit the needs. Changing the structure of an antenna to suit the need of the application is an engineering design problem. Here is where the concept of reconfiguration has been explored. If antennas are designed to be reconfigurable, then significant changes in the physical design need not be made to accommodate changes in the radiation pattern output that may be required [140].

The ability to change an antenna’s fundamental operating characteristics based on either electrical or mechanical means is termed reconfiguration. Ideally, reconfigurable antennas can alter their frequency of operation, impedance bandwidths, polarization, and other radiation parameters to accommodate changes in the output requirements [141]. Despite the huge cost that reconfigurable antennas pose, the concept of reconfigurability has gained momentum due to its wide range of applications in single element and array antenna scenarios. Small portable devices which use UWB range of frequencies may have to operate in harsh or unpredictable environments with weak signal strength in specific ranges of frequencies. In such cases, the reconfigurable antennas prove their mettle by switching from one frequency to another based on the conditions of operation. Reconfigurability requires complex control and feedback circuitry along with complicated fabrication procedures. Reconfiguration can be made possible in frequency, gain, or polarization. The main focus is to present frequency reconfigurable UWB notch antennas as they are the most widely used.

### 5.2. Frequency Reconfigurable Antennas

Frequency reconfigurable antennas have varied operations at different frequencies. A number of single frequency antennas can be replaced with a single reconfigurable antenna. Frequency reconfigurability is achieved using tuning mechanism, notching, or using switches [142]. Reconfigurable antennas can be integrated with electronic switches such as a PIN or Varactor diodes or switching through RF switches. Tunable materials with changing permittivity and varying height of the substrate can also be used for reconfiguration. PIN diodes provide the advantage of a low insertion loss, a good switching speed with a small size, and cheap cost. Lumped components such as PIN diodes, metal-oxide semiconductor field-effect transistors (MOSFETs), and RF MEMS (radio frequency microelectromechanical systems) switches can be used for performing switching. MEMS switches outperform PIN diodes and MOSFETs in terms of providing better isolation and consuming lower power. Tunable materials such as liquid crystals and ferrites can be used for reconfiguration as their dielectric constants can be changed with a change in voltage levels. This accounts for these materials changing their properties into other materials with varied permittivity and permeability [143].

In [144], electronic switches are placed on the slot of complementary split ring resonators (CSRRs), causing one to activate at a particular instant of time, causing band notching in a specific range of frequencies. On switching, the notch is created in another set of frequencies. In a second design, the notching appears when the switch is turned on and disappears when the switch is turned off. The authors of [145] use PIN diode switches to reject frequencies belonging to the Bluetooth and wireless LAN bands in UWB operation. Like [145], in [146], the rejection of WiMAX and WLAN bands is performed using PIN diodes. In [147], reconfiguration of the UWB antenna is achieved using a band stop filter. The filters are proposed using SRRs and CSSRs. Notches in four bands are obtained in [148] by shorting a slot of U-shape. The corresponding reconfiguration is achieved using an L-shaped slot for a wider band of resonance. In [149], two reconfigurable dielectric UWB antennas have been presented. Both these antennas achieve reconfigurability through rotation of the Dielectric Resonator (DR) with a stepper motor connected to it for activation.

The authors of [150] have presented the usage of two PIN diodes in a resonance cell to achieve reconfiguration of the UWB antenna by regulation of the capacitance using a varactor diode in the middle of the resonance cell. In [151], the band stop behavior is reconfigured to a bandpass behavior of the UWB antenna using PIN diodes with the CPW loaded S-SRRs. The authors of [152] present the design of a reconfigurable UWB antenna using stepped slots and five PIN diodes for switching in different stages.

The authors of [153] have presented a complex UWB reconfigurable antenna design that comprises three slots in the ground plane with parasitic strips on the feed resulting in seven states of operation. Five PIN diodes have been used to perform the switching between the seven stages of operation. References [154,155] have presented a G-shaped monopole UWB reconfigurable antenna with a PIN diode for switching and a cup-shaped UWB reconfigurable antenna with rejection in three bands using stubs and slots with three PIN diodes for switching, respectively. In [156], the authors have proposed the usage of hexagonal SRR metamaterial cells and switches for reconfiguration. In [157], the authors present a design of a band-notched UWB reconfigurable antenna which is achieved using varactor diodes placed between the split ring slots of the CSSRs used for notching. Finally, in [158], reconfiguration is achieved in the UWB notched antenna using biasing circuits of capacitors and inductors and PIN diodes for switching. Table 4 highlights the key features of the reconfigurable antennas presented above, along with the reasons for their reconfigurability.

The above section presented some prominent reconfigurable UWB notch antennas until 2020. This section has showcased that with the advent and popularity of reconfiguration, several antennas have been designed to suit the needs of band notching in the UWB range of frequencies. Despite the tradeoff of high complexity and preliminary cost of these designs, frequency reconfigurable antennas are gaining momentum due to the lasting feature of switching from one frequency to another, thereby reducing the need for multiple antennas to achieve the same purpose.

## 6. Conclusions

This article aims to present the trend of growth of UWB antenna technology from 2001 to 2020. The article describes the great potential of UWB technology in communication and the need to develop it further for varied applications. The first section of this article presented the UWB technology antennas with their various applications. It has been clearly observed that UWB technology can cater to several applications, ranging from ground-penetrating radars for disaster management to medical diagnostics and commercial applications such as USB dongles and mobile phones. Section 3 of this article presented the emergence of pure UWB antennas, which radiated in the range of 3.1 to 10.6 GHz. These antennas do not have notches in any frequency ranges. Due to the interference caused by the WLAN and WiMAX frequency bands, the concept of notching was introduced in UWB technology. Several UWB antenna designs have been presented in Section IV, which cause notching in various frequency ranges. Finally, Section V presented the concept of reconfigurable antennas in UWB technology, their need, and their advantages over conventional UWB technology. A review of antenna designs and their reason for reconfiguration has been highlighted in this section. UWB technology and antennas radiating in this range are here to stay. Therefore, this article aimed at presenting the birth, growth, and most recent trends in this field.

## Figures and Tables

**Table 1:** The emergence of UWB technology and its applications.

Ref. No.	Size (mm^2^)	Application of UWB Antenna	Gain (dBi)	Efficiency (%)	Antenna design	Advantage/Disadvantage	Reason for UWB Performance	Operating Frequency Range (GHz)
[8]	400	Mobile multiservice antenna	5	-	-	Advantage: Good performance for automotive application Disadvantage: Not suitable for high gain	The coplanar waveguide mode (CPW) and the coupled slot line mode (CSL).	0.6–5.0
[9]	600×76	Ground-Penetrating Radar	-	-	-	Advantage: Weak antenna ground interaction Disadvantage: Large size	Use a broad bandwidth feed to the dielectric circular waveguide’s hybrid HE11 (dipole mode).	1.0–6.0
[10]	171.5×163.3	Ground-Penetrating Radar	6	-	-	Advantage: Light weight Low radar cross-section Disadvantage: The balun hampers the performance of resistively loaded vee dipole (RVD)	The improved resistively loaded vee dipole (RVD) by curving the arms and modifying the Wu–King resistive profile.	0.5–8.0
[13]	45×60	WBAN applications	-	-	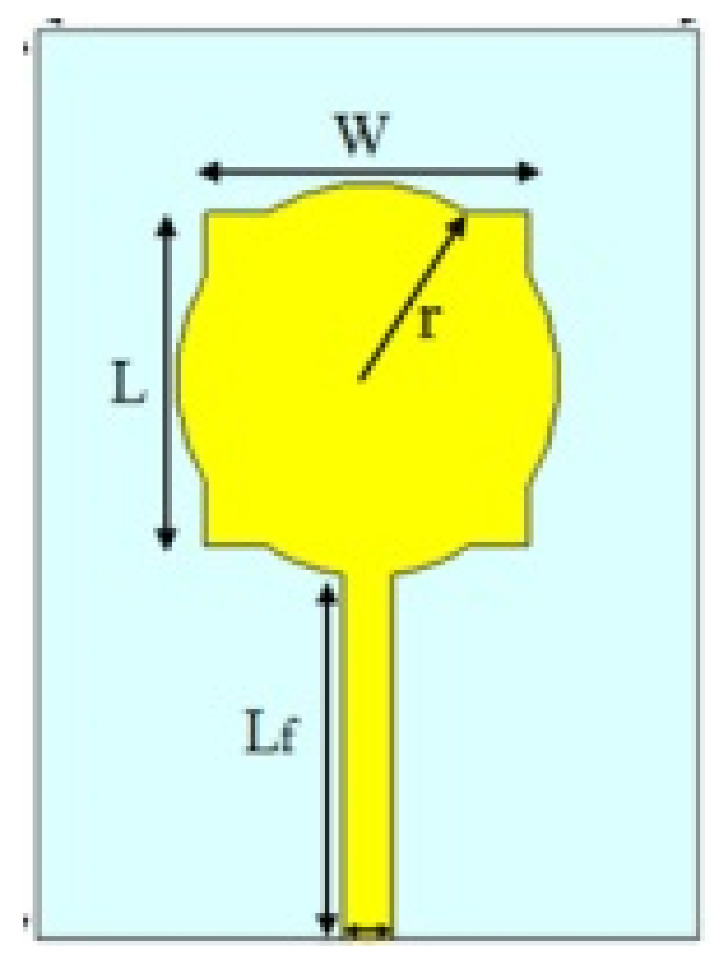	Advantage: Simple structure Disadvantage: Large size	A textile jeans substrate with copper tape for the radiator patch and the ground plane	2.2–17
[14]	36×48	Skin cancer detection using radiography	7	–	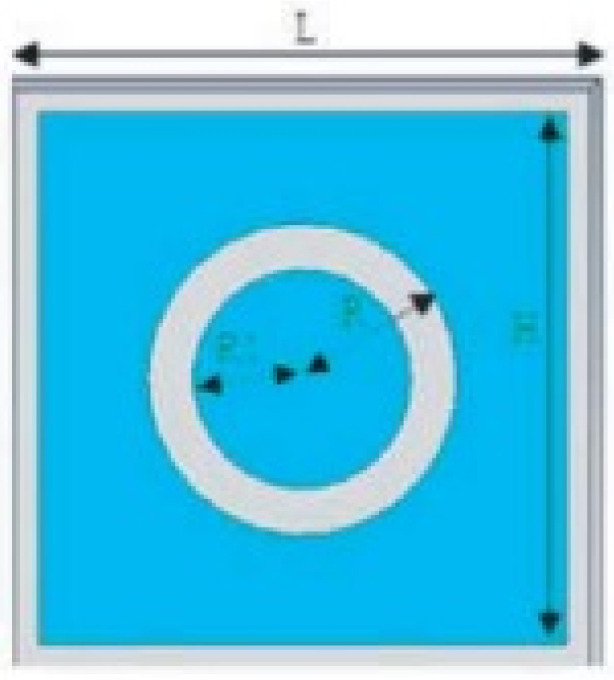	Advantage: Good stability Disadvantage: High SAR	Compact rectangular AMC antenna	8.2–13
[15]	80×67	Wearable applications	4.53	< 60%	-	Advantage: Shows good physical robustness Disadvantage: Less efficiency	Microstrip structure with 2 arc-shaped patches	3.7–10.3
[16]	50×45	Wearable applications	8	85	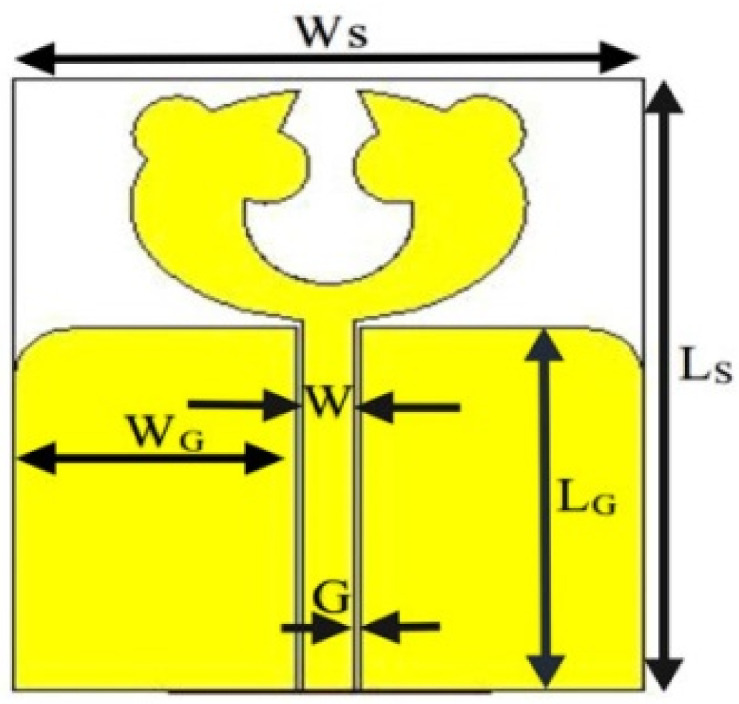	Advantage: Usage of metamaterial and array configuration gives increased gain and efficiency Disadvantage: Large size	Mu-negative metamaterial	7.2–9.2
[17]	40×45	Wearable Microwave Medical Imaging	2.9	-	-	Advantage: Easy to fabricate as it is made of flexible polyester Disadvantage: Complex design and possibility of coupling between the slots	Monopole antenna with triangular and parallel slots on the lower portion of the radiating patch	1.1–4.0
[19]	27×27	Wireless body area network (WBAN)	1.75–5.6	90.6	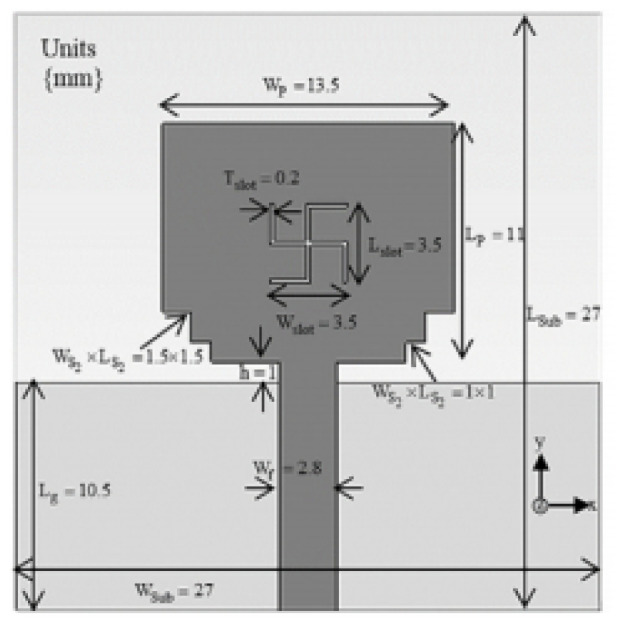	Advantage: Good gain and efficiency Disadvantage: The radiation pattern is not stable.	Partial ground plane with a slot in the patch and notches at the bottom	3.1–10.6
[20]	50×40	5G wireless communication	3.3	69	-	Advantage: Flexible antenna with good efficiency Disadvantage: Gain can be improved.	Cutting two slots in the ground plane	2.2–25
[21]	100×100	Wireless body area network (WBAN)	6	-	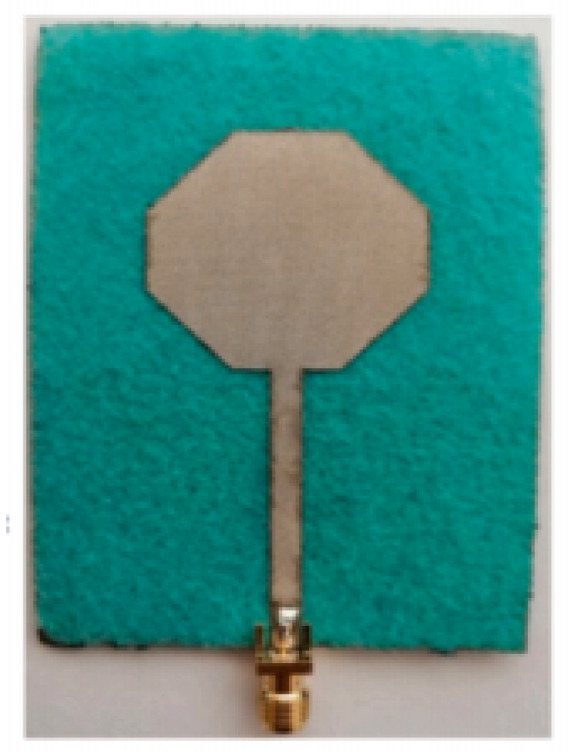	Advantage: Less thickness Low SAR Disadvantage: Large size	Use of rectangular ground plane and a circular parasitic patch at the back of the substrate	4.5–13
[24]	6×11	Wireless USB Dongle	3.2–4.6	94	-	Advantage: Good radiation characteristics Disadvantage: Ground plane length hampers impedance bandwidth	Two wide ended radiated arms and beveled feed	2.9–10.61
[30]	5×8	Mobile phone	-	88	-	Advantage: Good impedance matching and efficiency Disadvantage: Design complexity	Utilization of tapered structure	0.8–6
[40]	33.4×34.3	Human respiration and heart rate detection	14	-	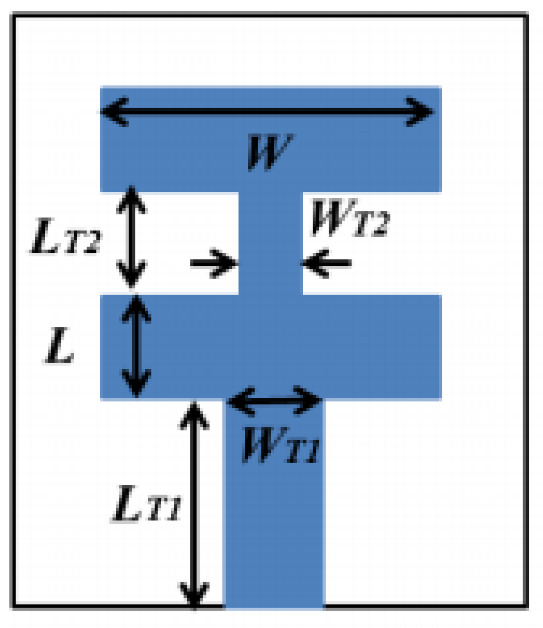	Advantage: High Gain antenna Disadvantage: The radiation pattern can be improved.	Doppler radar technique	10
[41]	32×28	Microwave Radiology Imaging (MRI) applications	6	-	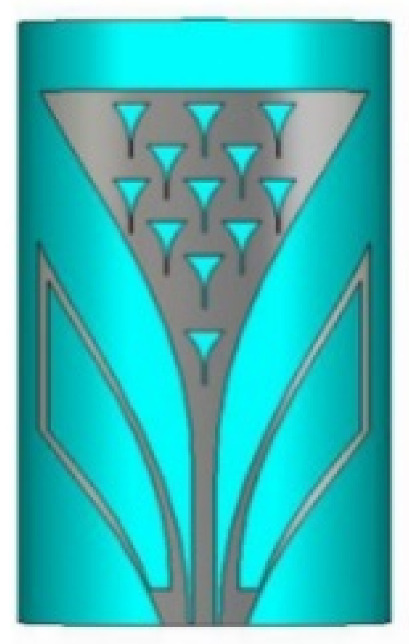	Advantage: Good performance for MRI applications Disadvantage: Large size	Slotted Triangular Flared (STF) patch	7.8–15
[43]	202×96	Detection of Void Inside Concrete Specimens	9.5	-	-	Advantage: It gives high-quality image of lost materials. Disadvantage: Large size	Use of tapered ground plane	1–30
[45]	48.6×48.6	Near-Field synthetic aperture radar (SAR) Imaging	3	-	-	Advantage: High impedance bandwidth and planar structure Disadvantage: Less gain	circularly polarized crossed dipole antenna with elliptical arms vacated by rotated elliptical slot.	1.6–7.2

Note: - indicates not applicable.

**Table 3:** The emergence of design of UWB notch antenna.

Ref. No.	Size (mm^2^)	Antenna Design	Gain (dBi)	Efficiency	Methodology	Notching Structure	Advantage/Disadvantage	Notch Frequency Range (GHz)
[82]	35×30	-	7	-	The rectangular aperture is designed by minimizing the aperture area and causing impedance matching.	Rectangular slot with T stub feed	Advantage: Good radiation pattern. Disadvantage: Gain decrease in second resonant mode due to cross-polarization decoupling	5–6
[83]	20×20	-	3.5	-	A parasitic strip to the lower end of the antenna with varied lengths causes notching.	Elliptic slot with three steps	Advantage: Good radiation characteristics and impedance matching. Disadvantage: Poor cross polarization	5.1–5.8
[85]	30×30	-	7	-	The concentration of current on the conductor’s outer edge and the fork-shaped strip causes band rejection in selected bands	T shaped slot in a fork-shaped strip	Advantage: Stable radiation pattern. Disadvantage: There is distortion in the incoming signal in the frequency band of interest.	5.1–5.8
[86]	29×30	-	4	-	Square ring resonator is used to achieve notching.	Octagonal slot with a rectangular stub for tuning	Advantage: Sharp frequency notch. Disadvantage: Radiation pattern can be improved.	5.2–5.9
[87]	26×26	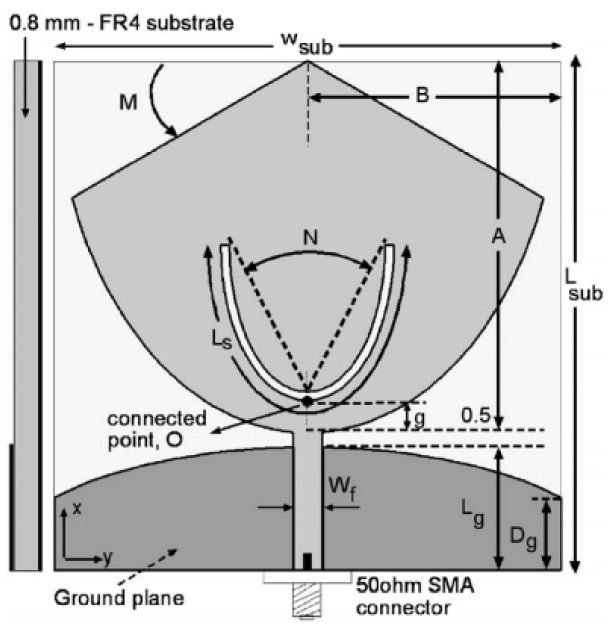	5	-	A semi-elliptical patch with dielectric material on both sides of the patch increases the effective patch, thereby increasing the bandwidth.	Two arc-shaped slots which are connected	Advantage: Good radiation pattern and good gain. Disadvantage: Complex design	5.1–5.9
[88]	22×24	-	5.4	-	A hexagonal patch with two slits is used for enhanced bandwidth and band rejection.	Hexagonal patch with two slits for band rejection	Advantage: Stable radiation pattern and good gain Disadvantage: Notch is not sharp.	5.1–5.8
[89]	38×38.5	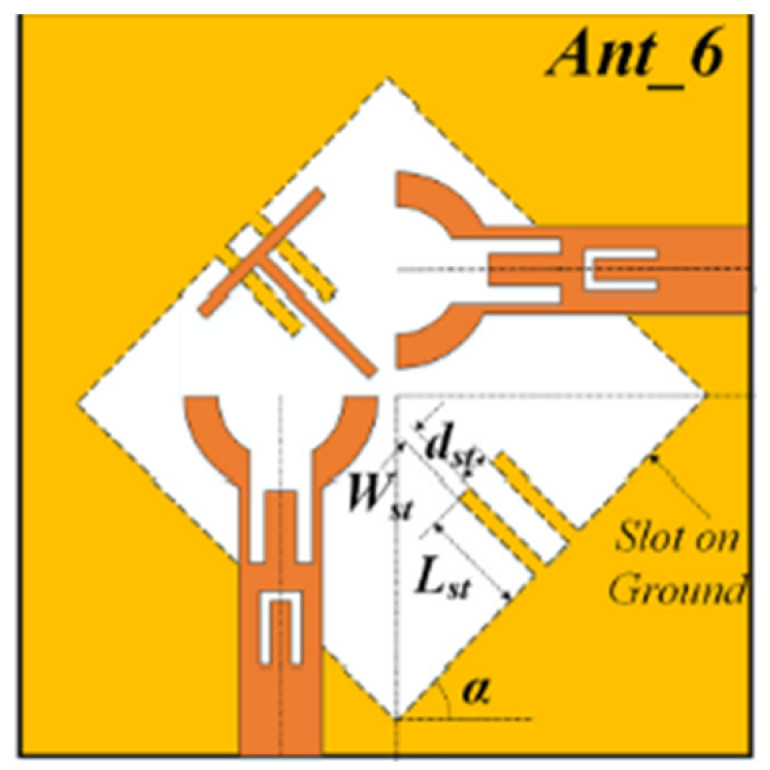	5	-	UWB antenna with U-shape slot and asymmetric rectangular slit	U-shape slot	Advantage: Stable gain and radiation characteristics Disadvantage: Complex structure	4.5–4.6, 7.0–7.4, 8.7–9.3
[90]	33×33	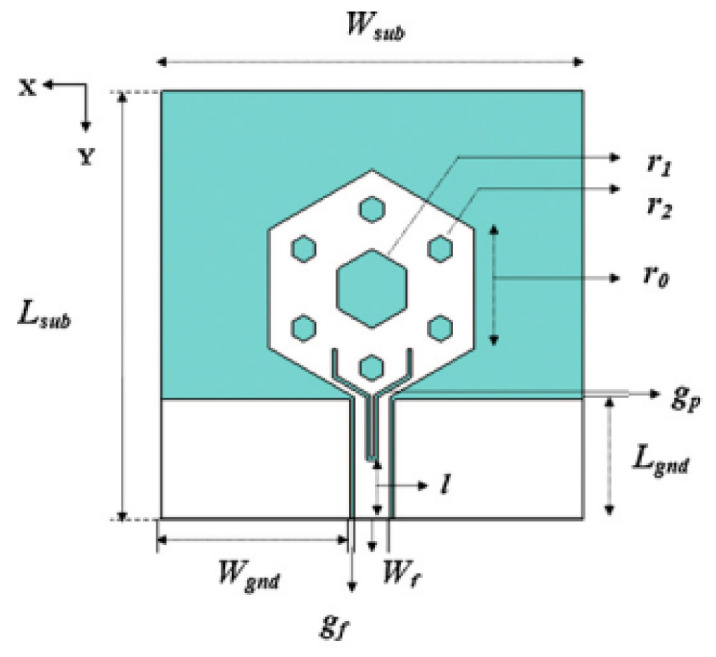	6	-	Application of fractal geometry in the hexagonal monopole	Y-shape slot	Advantage: Stable radiation pattern and good time-domain characteristics Disadvantage: Complex structure	5.1–5.8
[91]	33.5×28.5	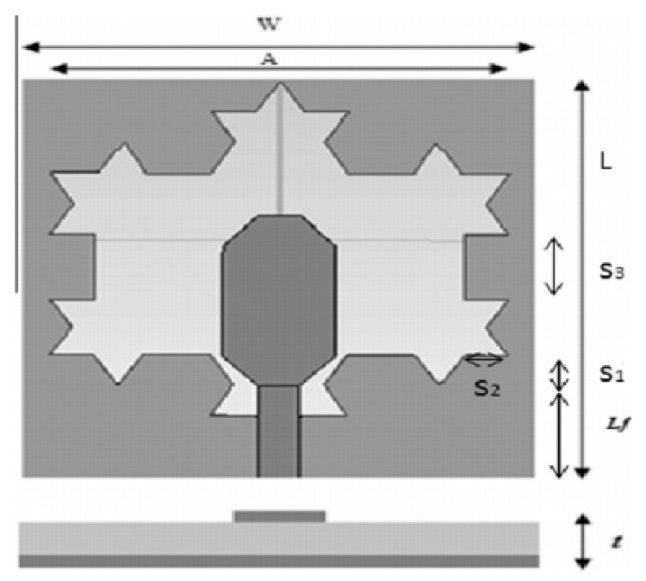	4	-	Use of Koch Snowflake iterative design	Snowflake slot	Advantage: Stable radiation pattern Disadvantage: Complex design	5.1–5.8
[92]	40×40	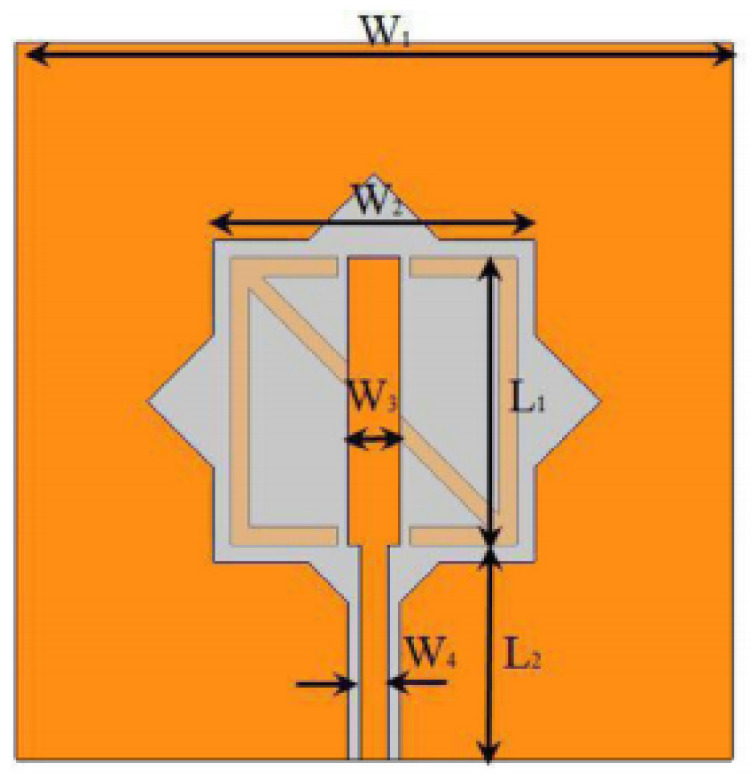	3.5	80	Use of fractal Koch with CPW feeding	Fractal Koch shape slot	Advantage: Circular polarization Disadvantage: Low gain at 1.5 GHz	1.5–5.4
[93]	50×50	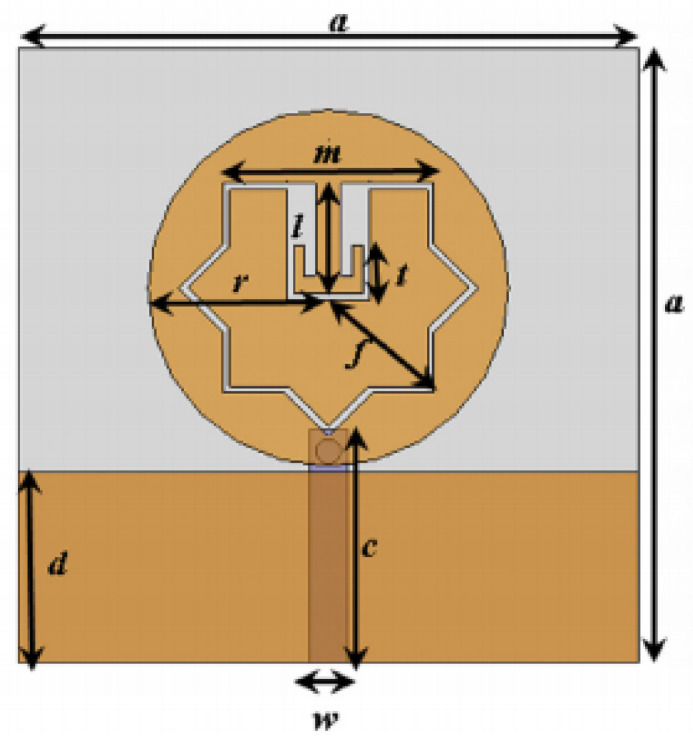	6.5	68	Fractal Koch with the stub of T-shape	Fractal slot	Advantage: Stable radiation pattern Disadvantage: Low efficiency	1.7–1.8, 2.2–3.1
[94]	20×20	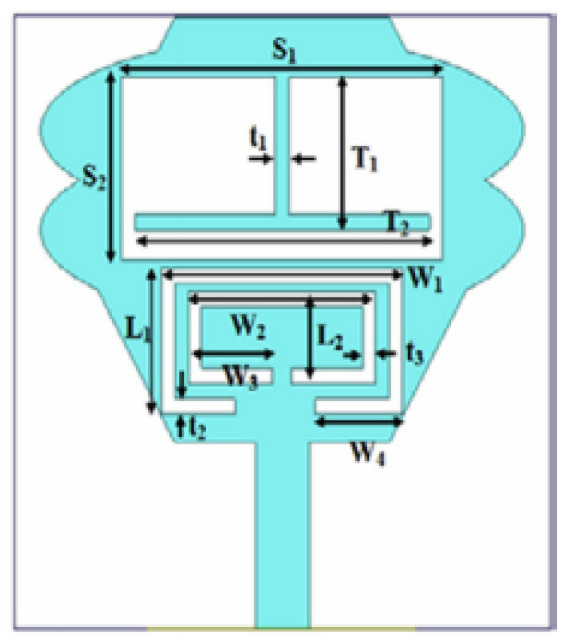	5.98	-	Use of two similar ellipses on a hexagon-shaped radiating patch	two C-shape slots	Advantage: Compact size Disadvantage: Radiation pattern is moderate.	3.1–3.8, 4.8–6.1, 7.2–7.8
[95]	39×39	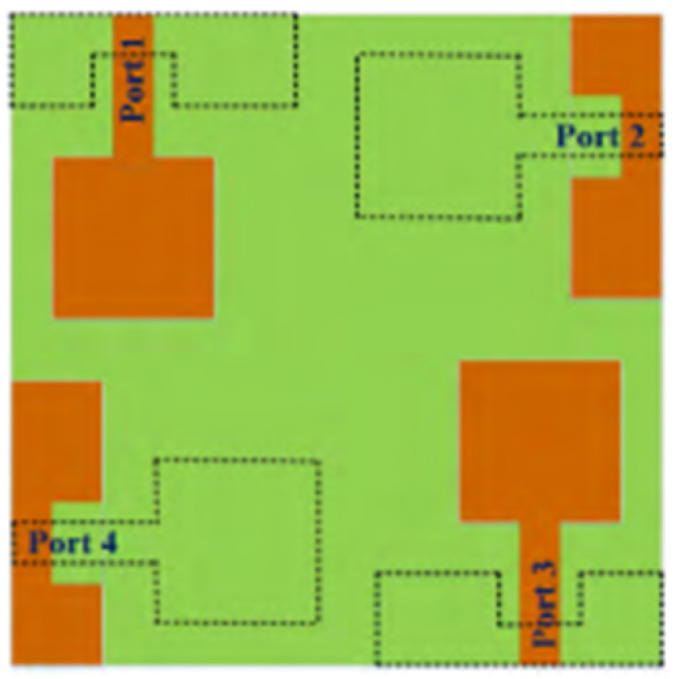	3.2	-	Use of decoupling structure, multi-slit, and multi-slot concepts	Multi-slot	Advantage: Low mutual coupling ( <−22 dB ) Low ECC Disadvantage: Gain can be improved.	3.2–3.7, 5.0–5.9, 7.0–7.9
[96]	19×30	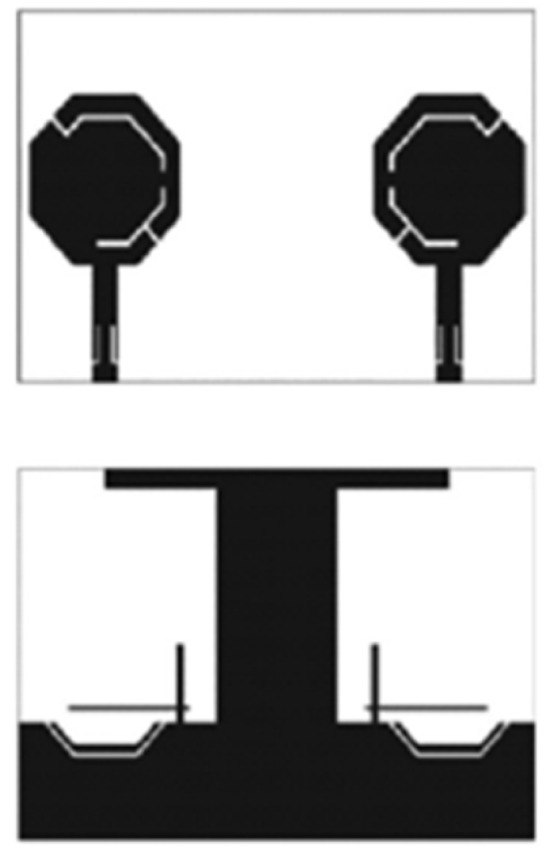	2.91	70–90	UWB antenna with T-shaped stub and open-ended slot	Open-ended slot	Advantage: Stable radiation pattern and low ECC Disadvantage: Low gain	4.3–5.9, 6.5–7.4
[103]	38×38.5	-	3.6	75	The offset microstrip-fed slot antenna	Rhombic slot	Advantage: Sharp notch, low mutual coupling, and low envelope correlation coefficient Disadvantage: Low efficiency	5.0–5.9
[104]	64×64	-	3	75	Use of orthogonal pairs of differential-fed elements and the irregular octagonal ground plane	Octagonal- shaped slot	Advantage: Wide bandwidth and low cross-polarization Disadvantage: Measured efficiency is lower than simulated efficiency	5–6.1
[105]	31×32	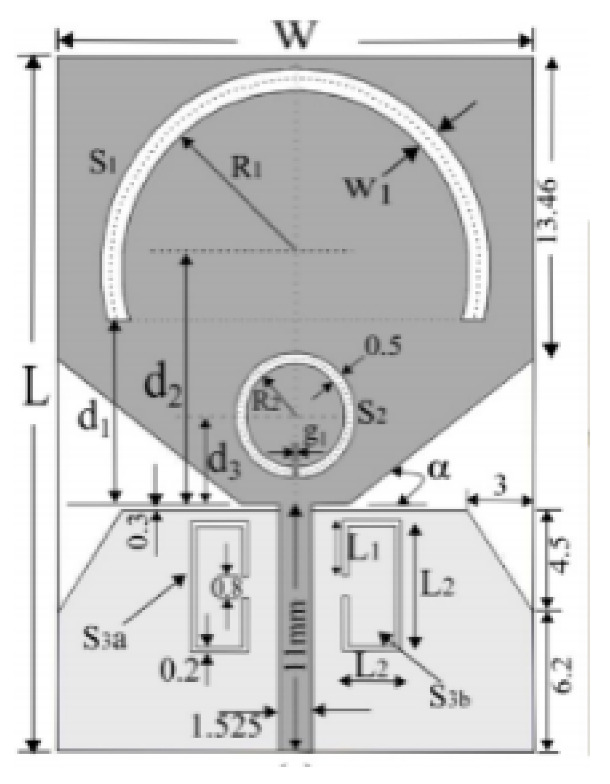	2.5	-	Use of two bevels in the upper edge of the rectangular ground plane	Round shaped slot, C-shaped slots	Advantage: Stable gain and sharp notch band Disadvantage: Unstable radiation pattern	3.3–3.7, 5.1–5.8, and 7.1–7.7
[106]	30×30	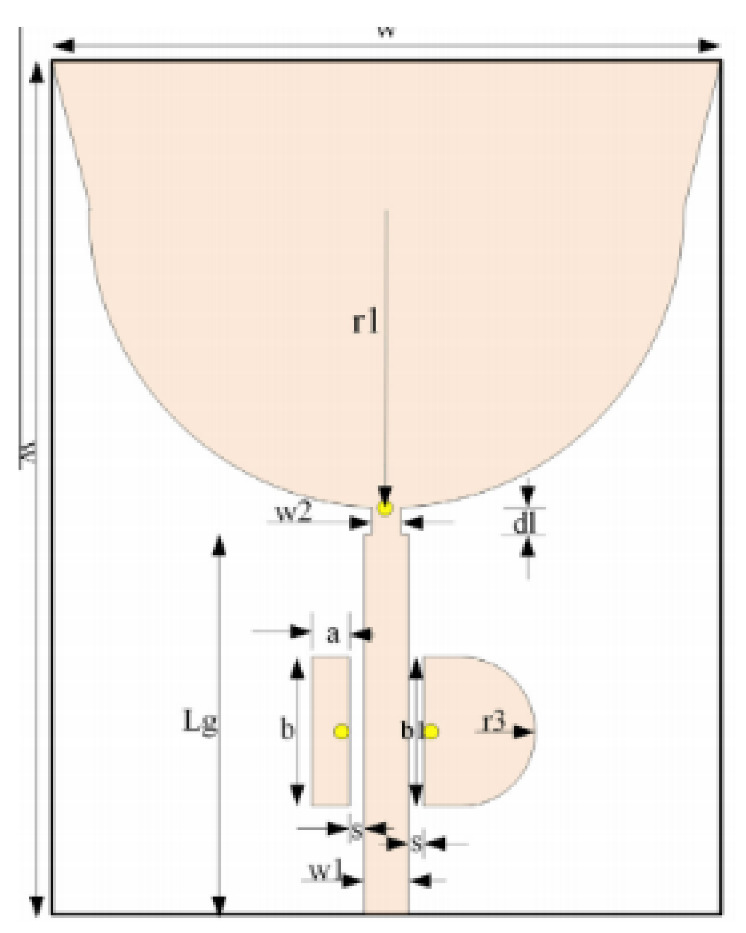	5	-	Monopole patch antenna consists of semicircular radiator truncated with a trapezoidal structure and a ground of rectangular shape	Inverted-L slots	Advantage: Simple structure and easy to fabricate Disadvantage: Unstable radiation pattern	3.3–3.8, 5.1–5.9, and 8.0–8.7
[107]	26×30	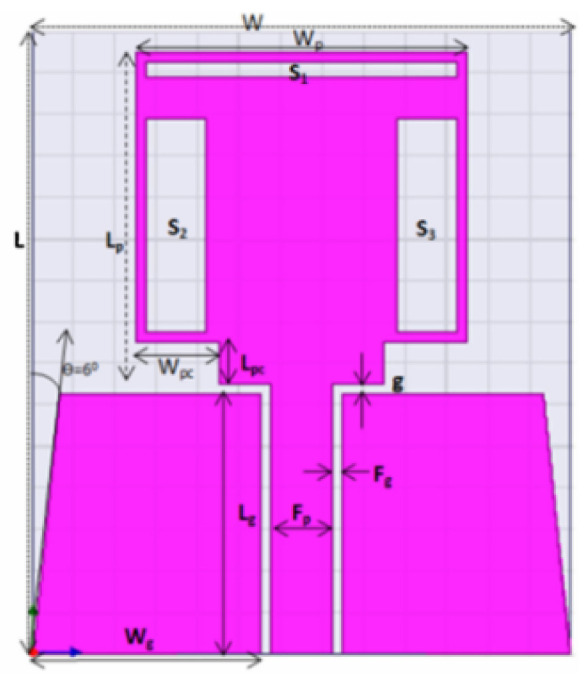	4.9	90	CPW fed printed monopole antenna	Three rectangular slots	Advantage: Simple structure and compact size Disadvantage: Gain is not flat throughout the operating band.	5.1–5.8 and 7.2–8.3
[108]	50×40	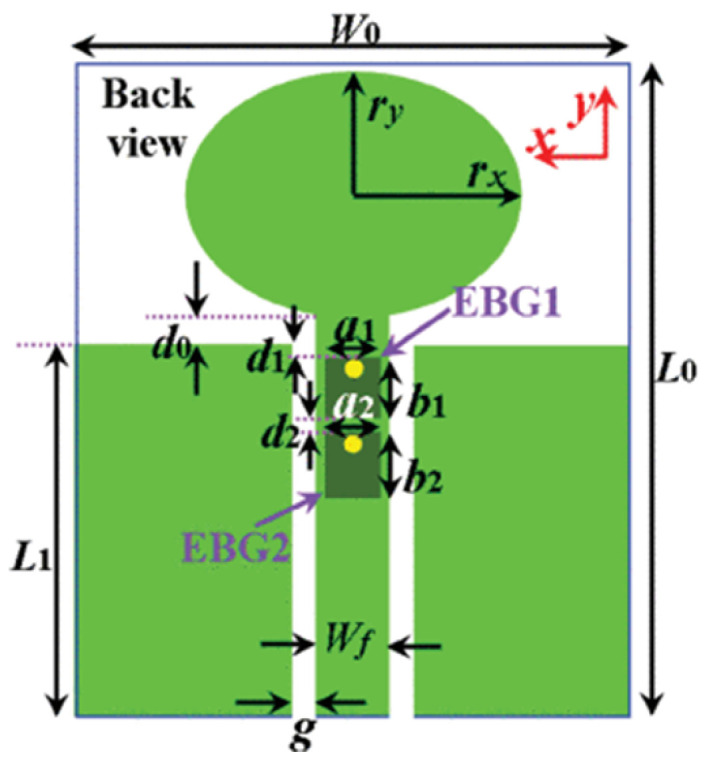	5.1	97	CPW fed UWB antenna with EBG structure	Rectangular slot on the ground plane	Advantage: Good gain and better efficiency Disadvantage: Large size	5.1–5.8, 7.1–7.7
[109]	104×100	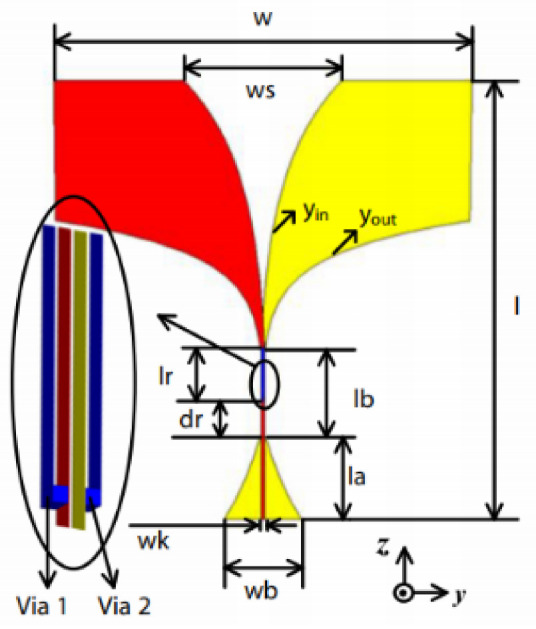	6.5	-	Antipodal Vivaldi antenna with resonant parallel strip	Resonant parallel strip	Advantage: Stable radiation pattern Disadvantage: Large size	3.3–3.6
[110]	30×30	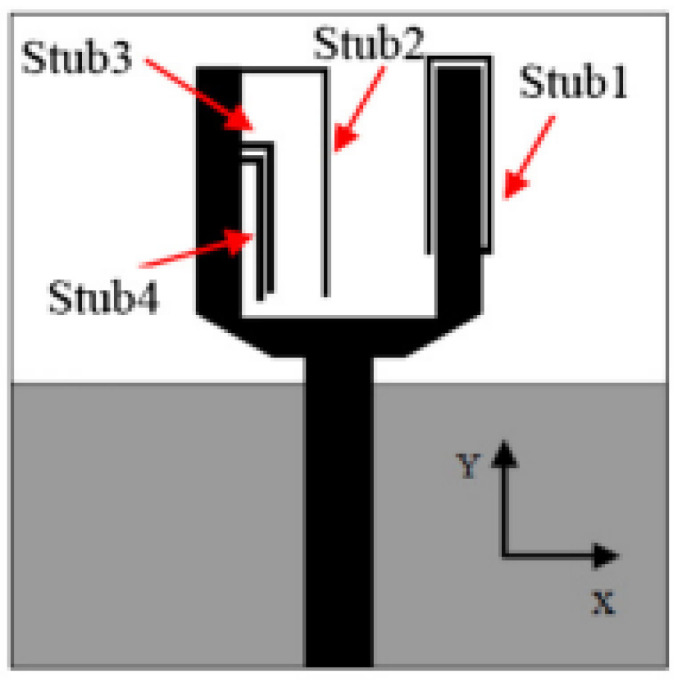	4.8	55	A quasi-U-shape antenna with four stubs and a stepped ground plane	stepped slot	Advantage: Stable radiation pattern Disadvantage: Less efficiency	4.9–5.4, 5.6–5.9
[111]	35×30	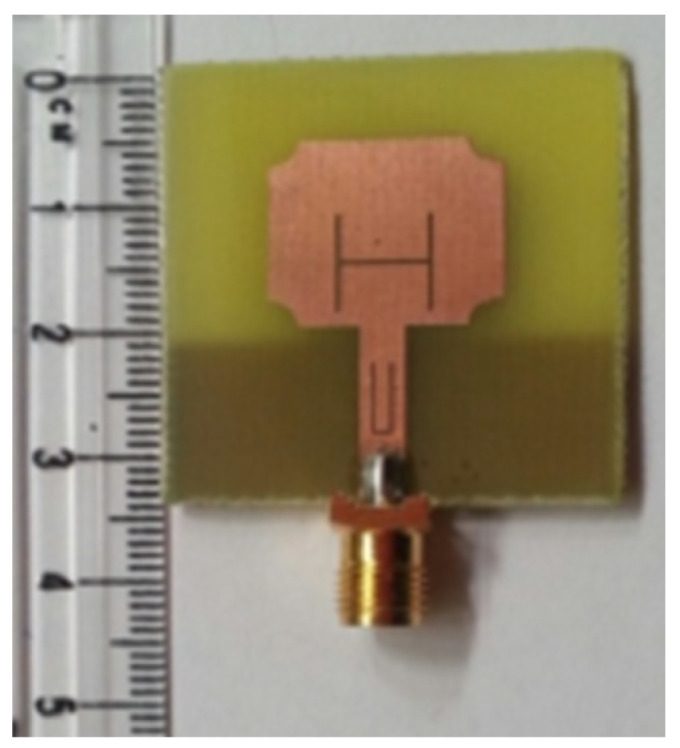	6	-	Rectangular patch antenna with H-slot on patch along with a feed with a U-slot	H-shaped and U-shaped slot	Advantage: Good gain Disadvantage: Moderate radiation pattern	5.1–5.8, 7.2–7.7, 7.9–8.4
[112]	36×38	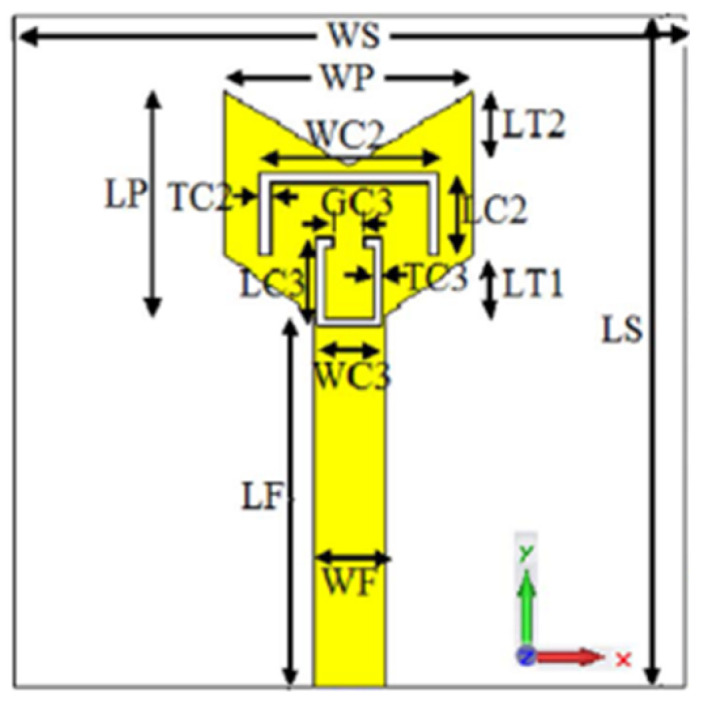	6	-	Y-shaped antenna with slot of inverted U-shape along with slot of C-shape on radiating patch and ground plane	inverted U-shaped and a C-shaped slot	Advantage: Omnidirectional radiation pattern Disadvantage: phase response is little distorted.	3.4–4.0, 5.1–5.9, 6.7–8.0, 8.3–9.1, 9.3–10.6
[113]	32×24	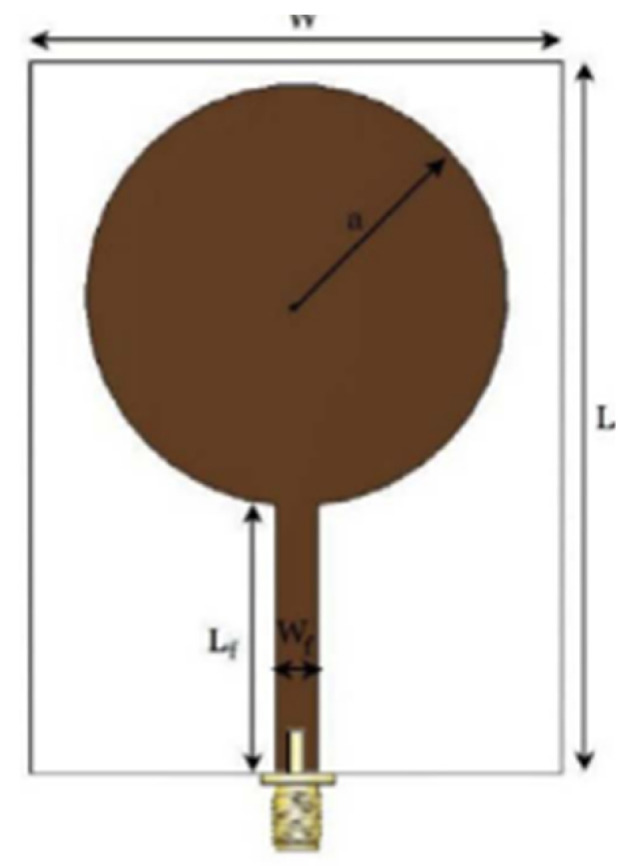	4.4	95	Circular monopole antenna with DGS and resonators of inverted-U and the iron shape on the substrate	Rectangular slot	Advantage: Compact size Disadvantage: Distortion in the radiation pattern	5–5.4, 7.8–8.4
[114]	20×40	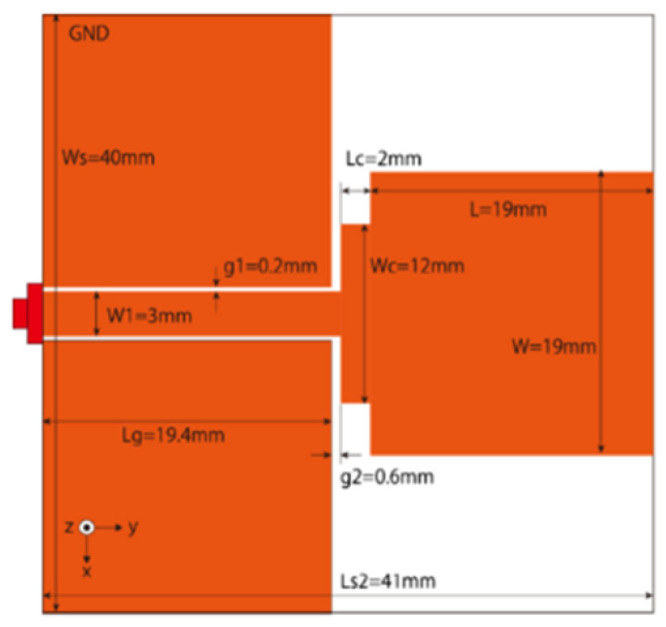	5	-	CPW-fed UWB antenna with sharp bandstop filter (BSF)	Bandstop filter (BSF)	Advantage: Omnidirectional radiation pattern Disadvantage: Distortions at higher frequency in E plane	5.7–5.8, 8.0–8.4
[115]	24×36	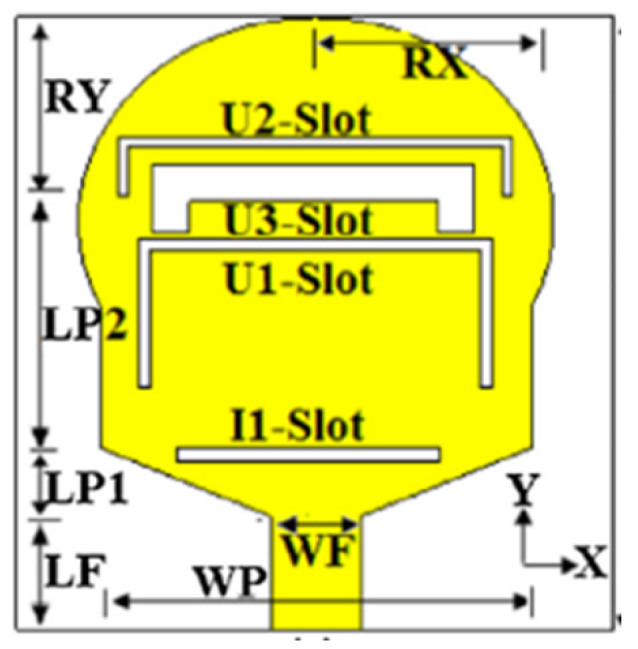	6.15	-	Monopole ellipzoidal antenna with three slots of inverted U-shape and one slot of I-shape slots	Inverted U-shape slot, I-shape, and rectangular slot	Advantage: Omnidirectional radiation pattern and linear phase Disadvantage: Complex design	3.2–3.9, 4.3–5.0, 5.5–6.6, 7.9–9.3
[116]	64×45	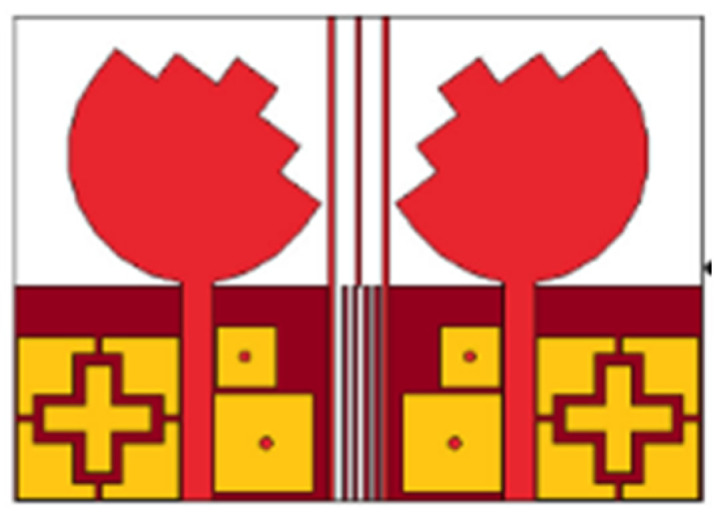	5.8	-	Antenna with two Mushroom and One Uniplanar EBG Structures	Electromagnetic Band Gap (EBG) structure	Advantage: Good diversity parameters obtained Disadvantage: Less isolation	3.3–3.6, 5–6, 7.1 –7.9
[117]	25×25	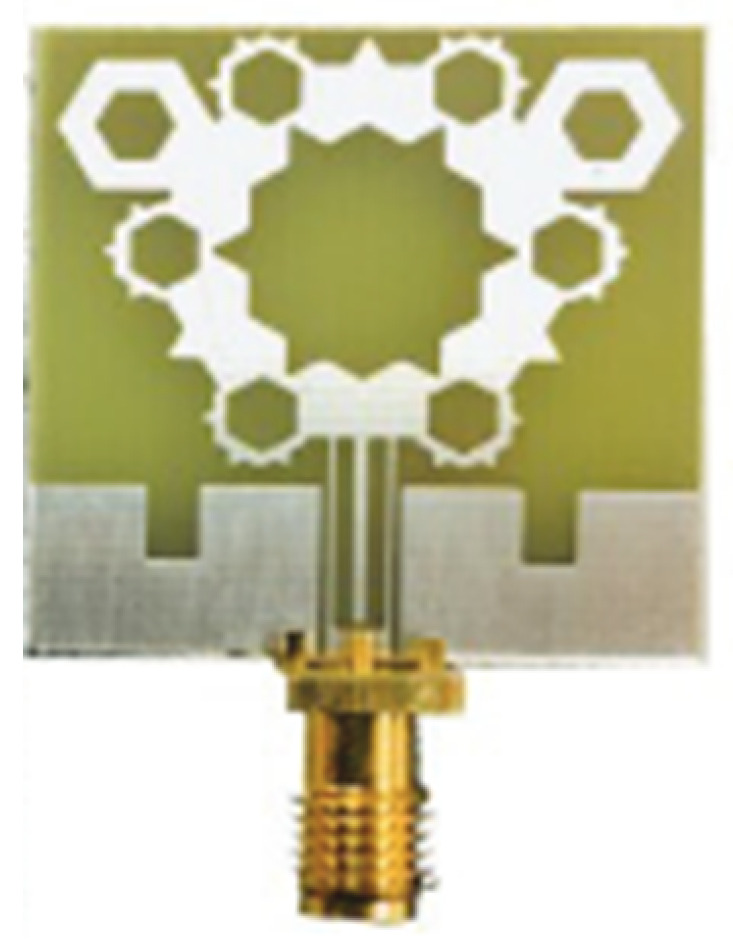	-	-	CPW-fed Hexagonal Fractal Antenna with design of the grooves in the ground plane and use of fractal geometry and Notch filters.	Hexagonal and U-shape slot	Advantage: Omnidirectional radiation pattern and good return loss <−10 dB Disadvantage: Complex structure	5.1–5.9
[118]	32×16.5	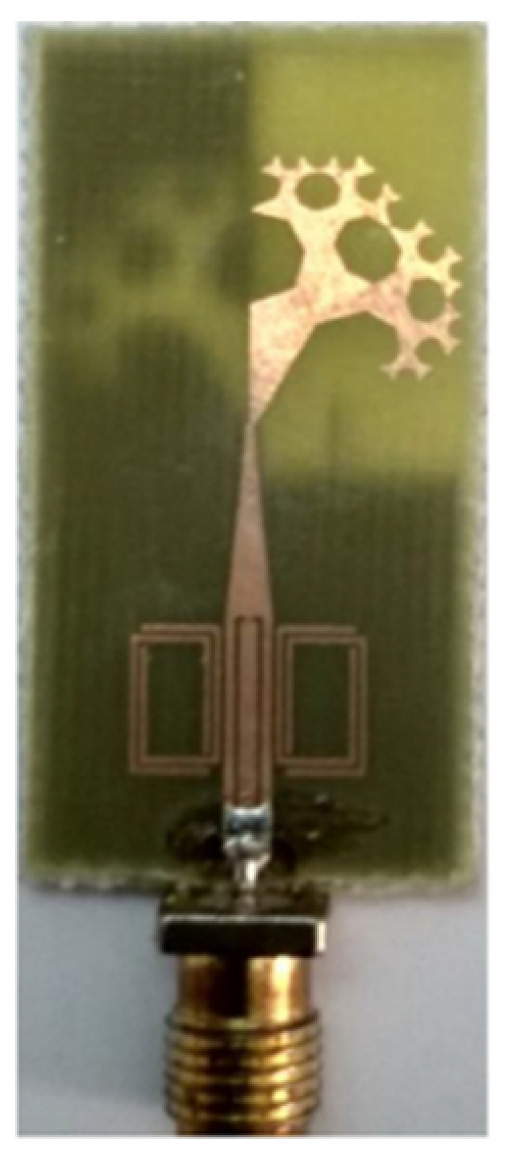	2.2	96	A QSCF antenna with loading slot and stub	U-shaped slot	Advantage: omnidirectional radiation pattern Disadvantage: Low gain	3.3–3.8, 5.1–5.8, 7.2–8.3
[119]	38.5×38.5	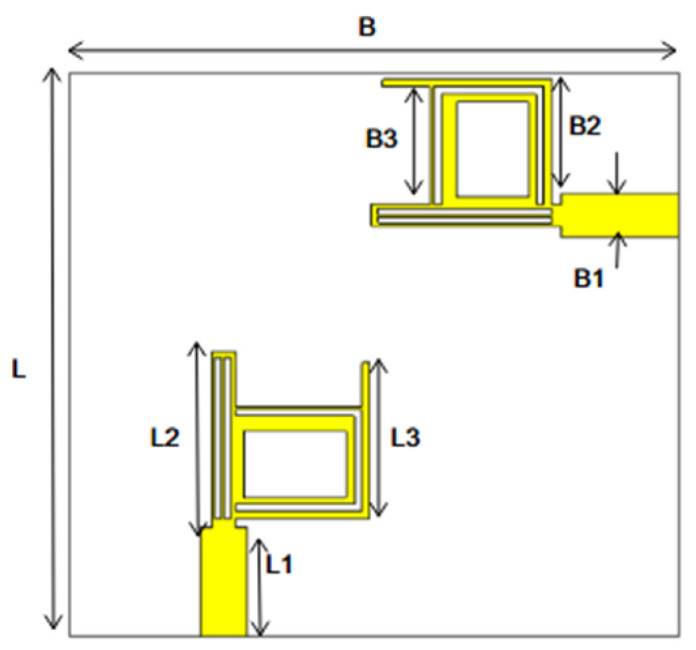	6	-	inverted-A Monopole UWB antenna with defected ground structure (DGS).	Rectangular slot	Advantage: High isolation and low diversity performance Disadvantage: More number of multipath	3.9–4.2, 5.1–5.8
[120]	20×14	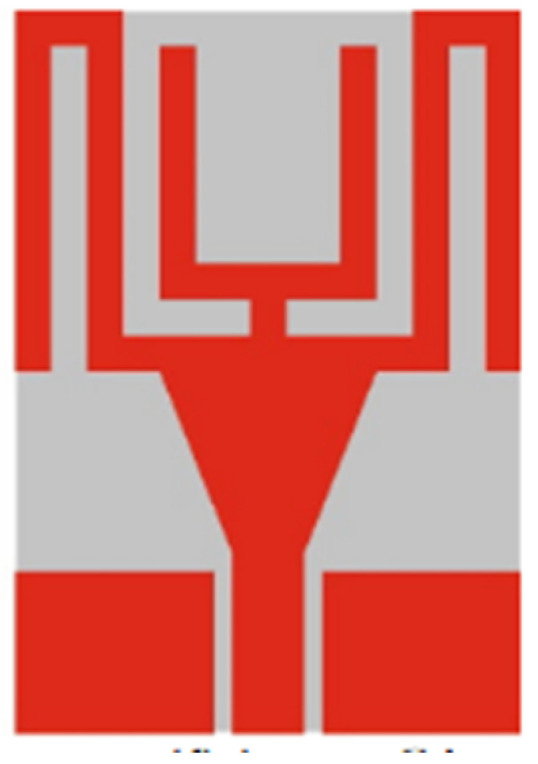	5	80	A double fork-shaped CPW fed structure and a simple symmetrical inverted L-shaped stub resonator	Inverted L-shaped stub resonator.	Advantage: Small size Omnidirectional radiation pattern Disadvantage: At 20 GHz radiation pattern is less bidirectional in the E-plane	3.4–3.8, 4.5–5.5
[121]	34.6×22	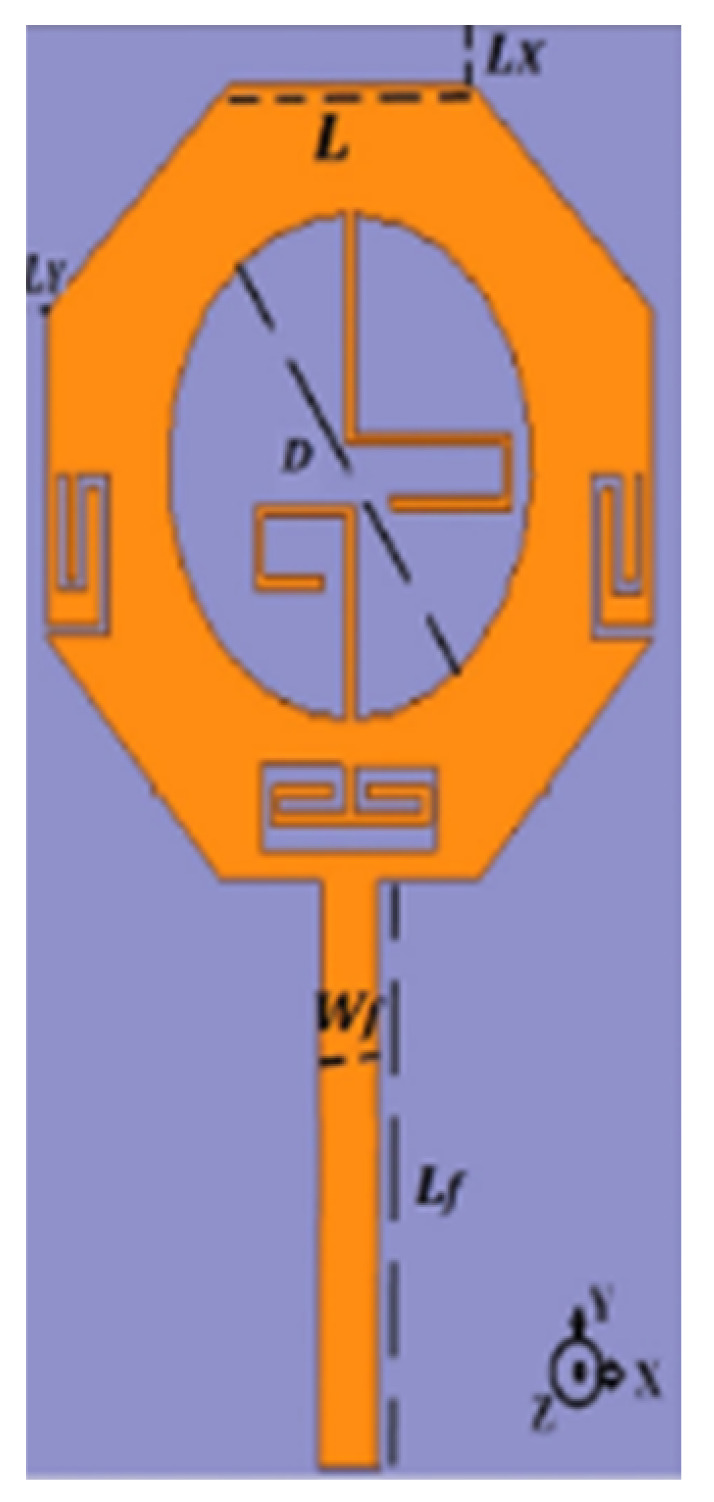	4	-	UWB monopole antenna with the defective ground structure of P-shape resonators, slot resonator pair, a stub of T-shape, and conductor plane of two slits.	Resonator, slot, and slit	Advantage: Sharp notched band and good time-domain characteristics Disadvantage: Complex design	3.3–3.6, 5.1–5.3, 5.6–5.9, 7.2–7.6, 7.8–8.2, 9.2–9.7
[128]	26×26	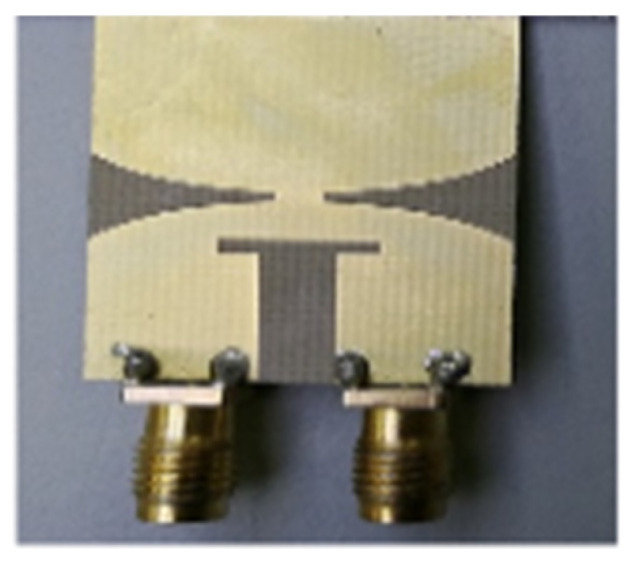	5	-	UWB Vivaldi antenna with two microstrip feeding lines, T-shaped slot on the ground plane, and two split-ring resonators (SRR)	T-shaped slot	Advantage: Very low ECC and stable gain Disadvantage: Isolation can be improved	5.3–5.8, 7.8–8.5
[129]	25×26	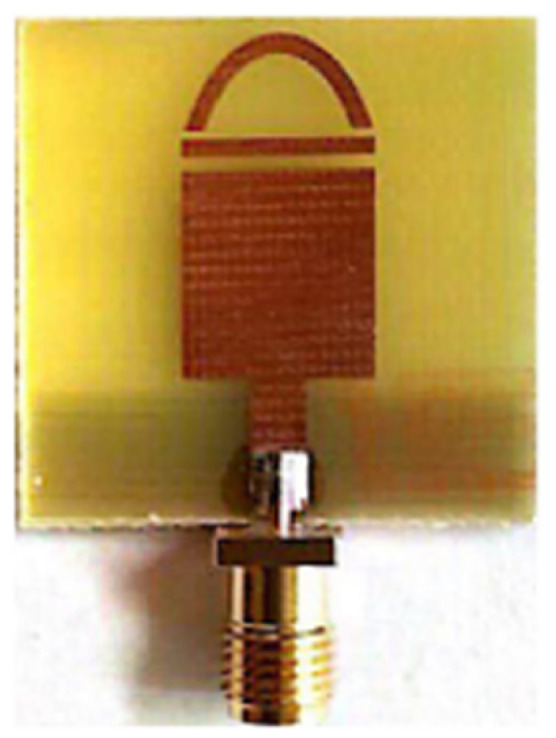	6.2	70	Antenna with a rectangular patch and a partial ground plane	open-loop resonator	Advantage: Compact size and good gain Disadvantage: Less efficiency	3.3–3.8, 3.7– 4.2, 5.1–5.8, 5.5–5.9
[130]	38×40	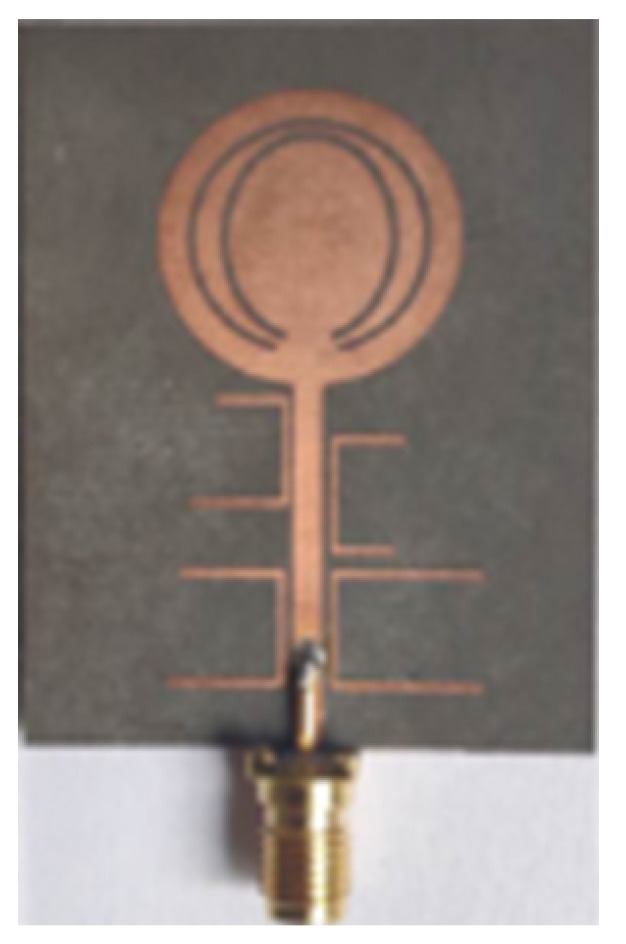	4	-	Antenna with elliptic radiator and a rectangle ground plane using ESRR and RSRR with U-shaped parasitic strip	elliptic split ring resonator (ESRR) and a round split ring resonator (RSRR)	Advantage: Compact size, wider bandwidth, and more notched bands Disadvantage: Distortion in radiation pattern	2.9–3.3, 3.7–3.8, 4.4–4.5, 5.3–5.5, 7.0–7.3, 7.5–8.0
[131]	45×31	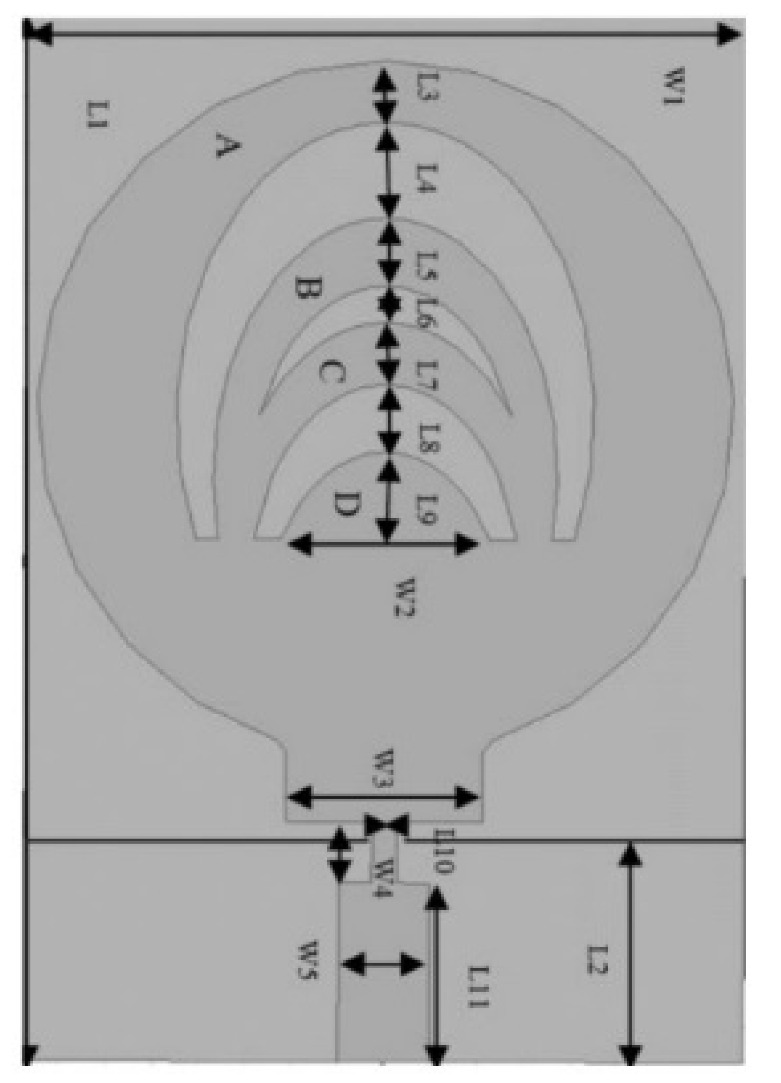	5.2	-	Metamaterial based ring resonators	Double-slotted ring resonators (SRR), circular (C-SRR), and square (S-SRR)	Advantage: Multiple band frequencies rejection and stable radiation patterns Disadvantage: Complex structure	4.6–5.4
[132]	24×16	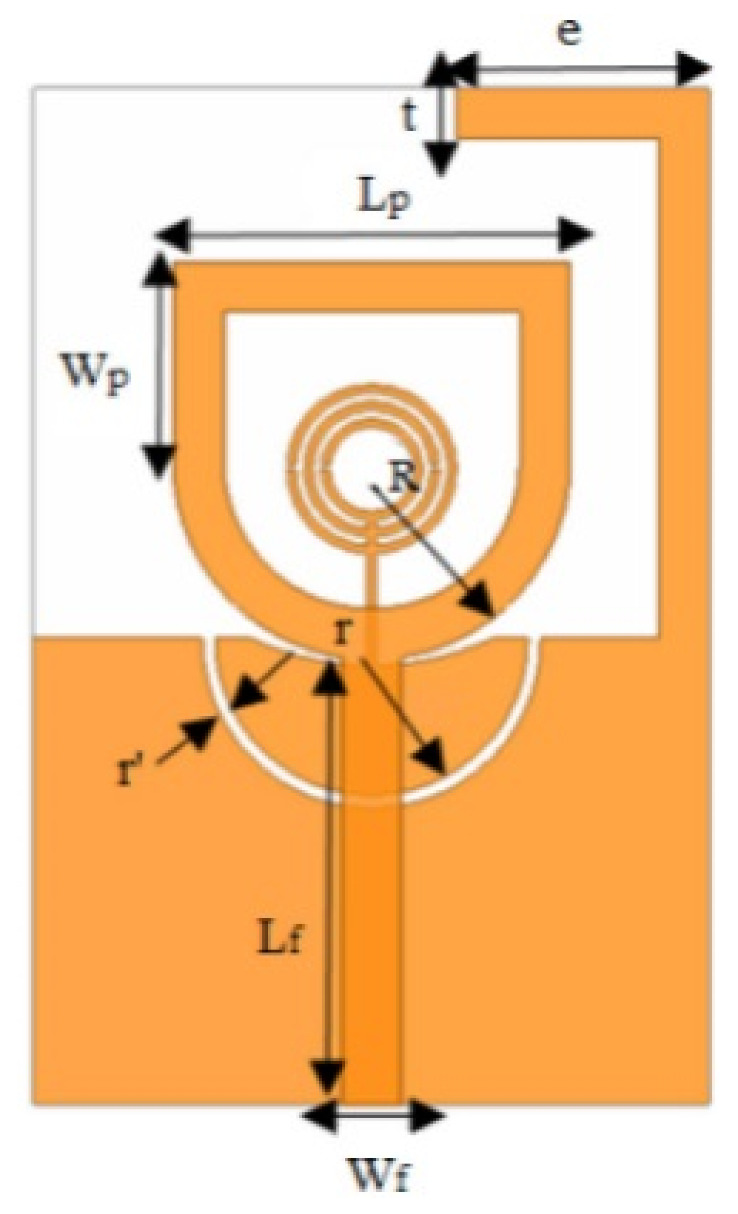	-	-	Metamaterial based split ring resonator	SRR on the ground plane	Advantage: Compact size and simple structure Disadvantage: Disturbance in the radiation pattern	5–6
[133]	40×30	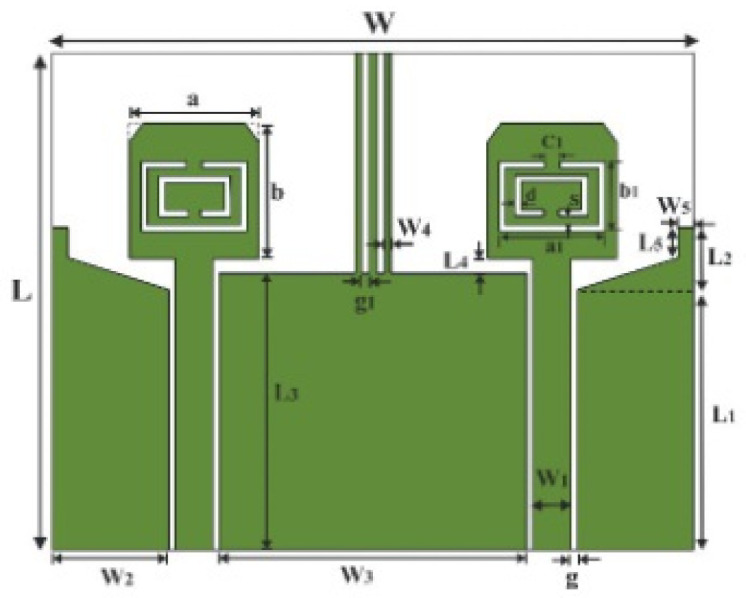	9.94	58	Metamaterial based split ring resonator	A complementary split-ring resonator (CSRR) and the split ring resonator (SRR)	Advantage: Good isolation, low ECC, and stable gain Disadvantage: Low efficiency	3.4–3.9, 5.7–6.2
[134]	50×50	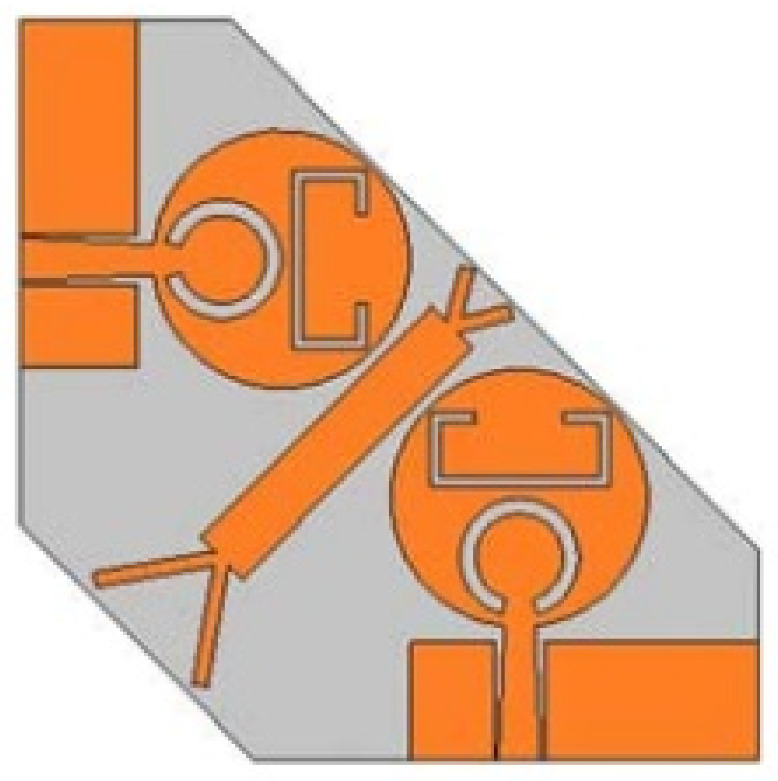	4.71	-	Metamaterial based split ring resonator	Annular SRR slot and a rectangular SRR	Advantage: Better isolation and good gain Disadvantage: Large size	3.3–3.9, 4.7–5.5
[135]	20×26	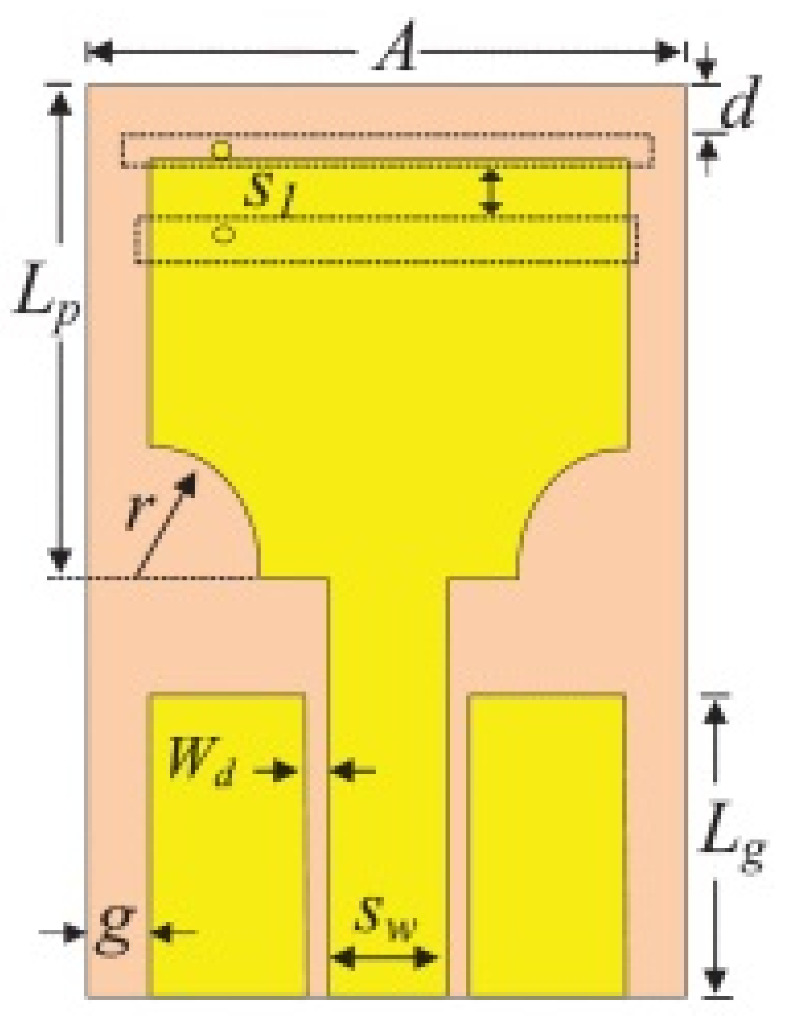	3.86	-	EBG and Metamaterial based split ring resonator	2 split-ring resonators (SRR)	Advantage: Compact size and stable gain Disadvantage: Poor isolation	3.4–3.9, 5.1–5.8, 7.2–7.7
[136]	29×26	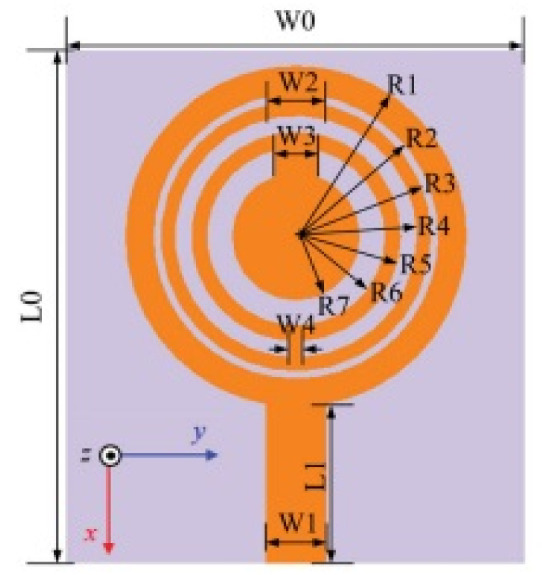	3.47	-	Annular defected ground	Multi resonant metamaterial cell	Advantage: Compact size and simple structure	–
[137]	67×67	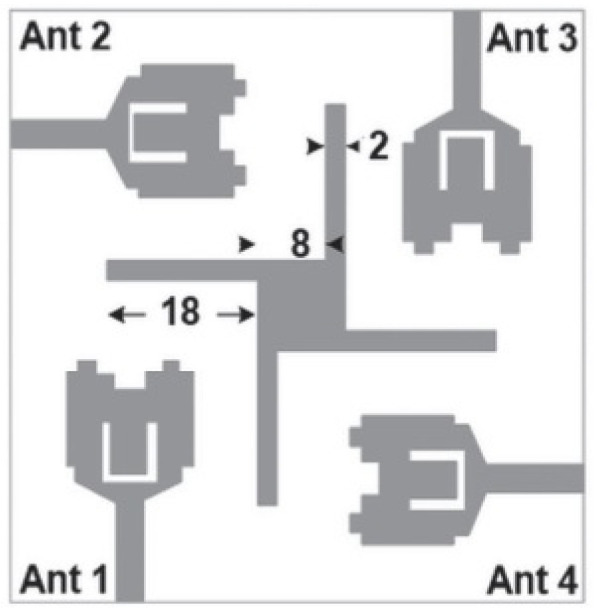	8	79.4	Parasitic decoupler	U-shape slot on the patch	Advantage: Higher gain, Good isolation Disadvantage: Less efficiency	4.2–5.8
[138]	24×18.5	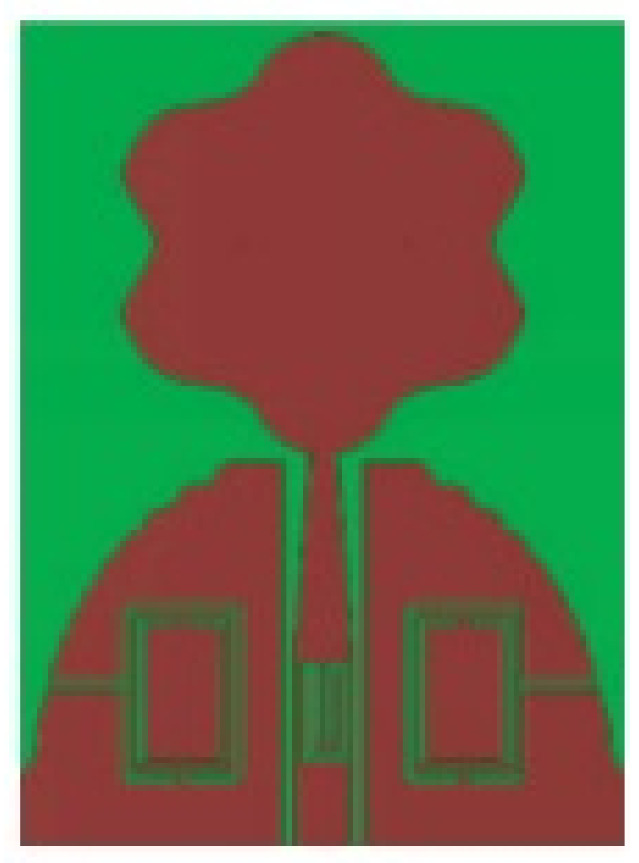	4	-	Metamaterial based split ring resonator	Split ring resonator (SRR) and U-shape slot	Advantage: Simple structure and wide band Disadvantage: High dispersion	3.3–3.8, 5.1–5.8, 7.2–8.3
[139]	22×26	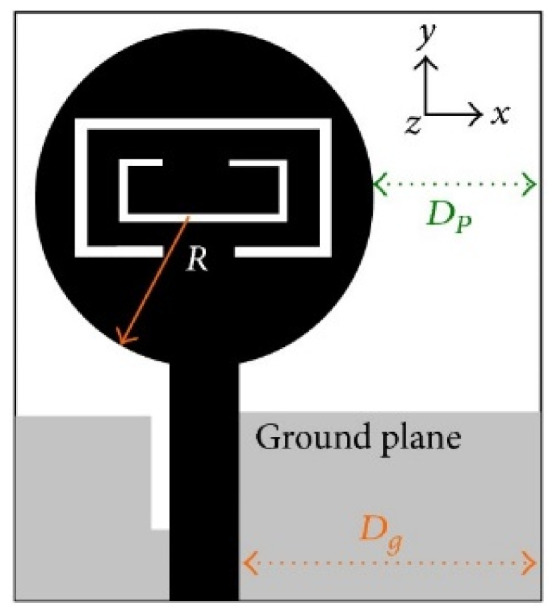	4	-	Metamaterial based complementary split-ring resonator	Slotted complementary SRRs structures	Advantage: Compact size, simple structure, and wide band Disadvantage: Gain is not constant in the operating band.	4.8–5.7

Note: - indicates not applicable.

**Table 4:** The emergence of design of frequency reconfigurable UWB notch antenna.

Ref. No.	$\mathrm{Size}\;(mm^{2})$	Antenna Design	Gain (dBi)	Efficiency (%)	Advantage/Disadvantage	Frequency Reconfiguration Mechanism	Design Methodology	Operating Frequency (GHz)
[144]	30×30	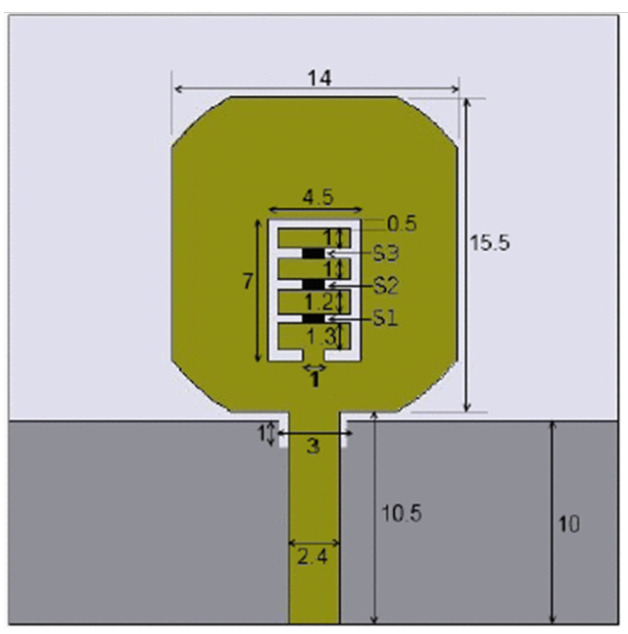	3.12	-	Advantage: Wider band notch Disadvantage: Less gain	Electronic switches	Complementary split-ring resonators (CSRRs)	3.8–9.5
[145]	17×17	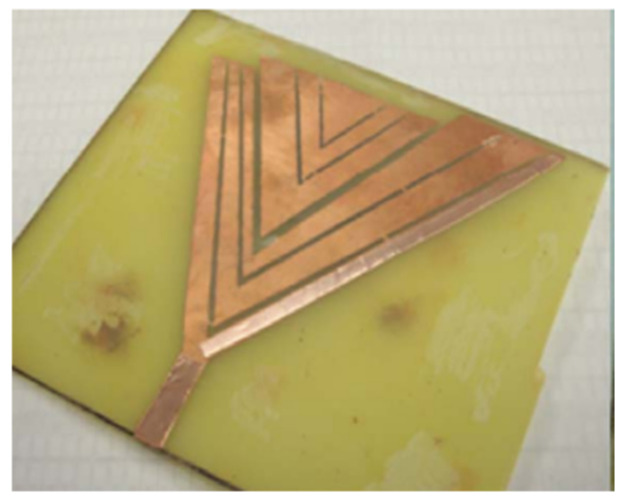	16	70	Advantage: Compact size and good gain Disadvantage: Less efficiency	PIN diode	Monopole of V- shape and electromagnetic band-gap structure with U-shaped slot	0.6–30
[146]	28×30	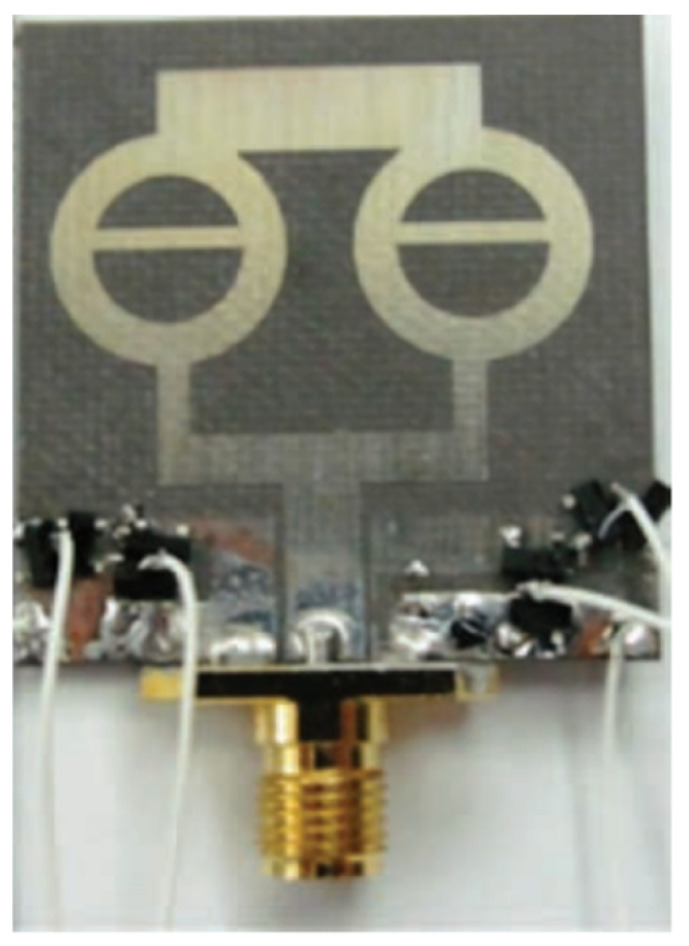	-	-	Advantage: Each notch band can be controlled separately. Simple design Disadvantage: Complex biasing circuit	PIN diodes	A CPW fed UWB antenna using a defected ground-plane slit	2.8–10.6
[147]	25×20	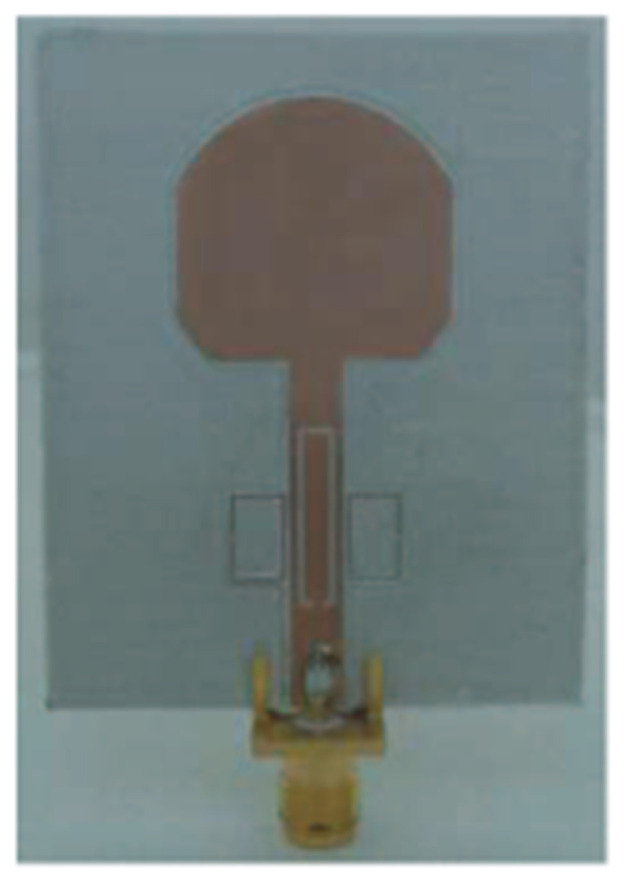	5	-	Advantage: More unlicensed users can operate since it dynamically rejects a band. Disadvantage: The efficiency of the proposed antenna needs to be further explored.	RF switches	Filter antennas using CSRRs and SRRs	2.1–7.5
[148]	30×31	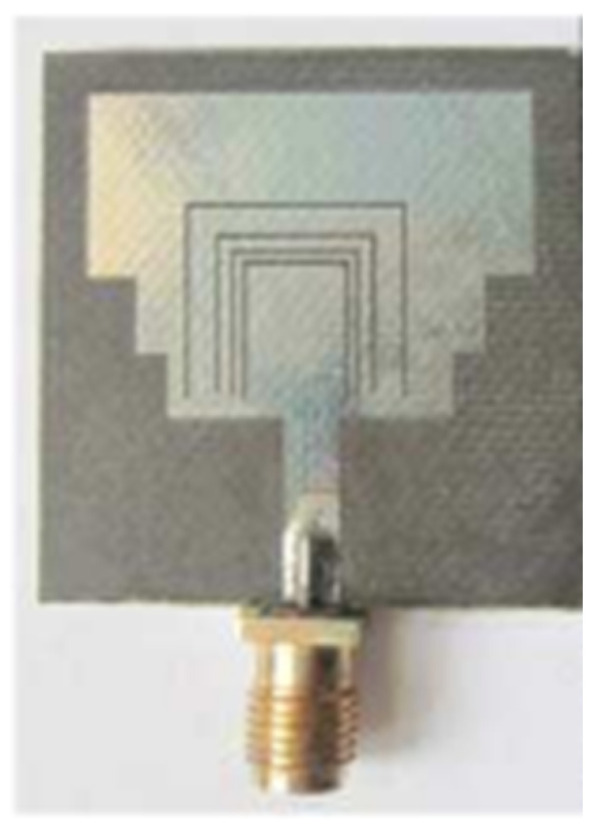	4.5	-	Advantage: Simple structure and constant group delay Disadvantage: Distortion during pulse transmission	RF switches	Shorting the U-shaped slots on the patch	2.4–10.8
[149]	50×44	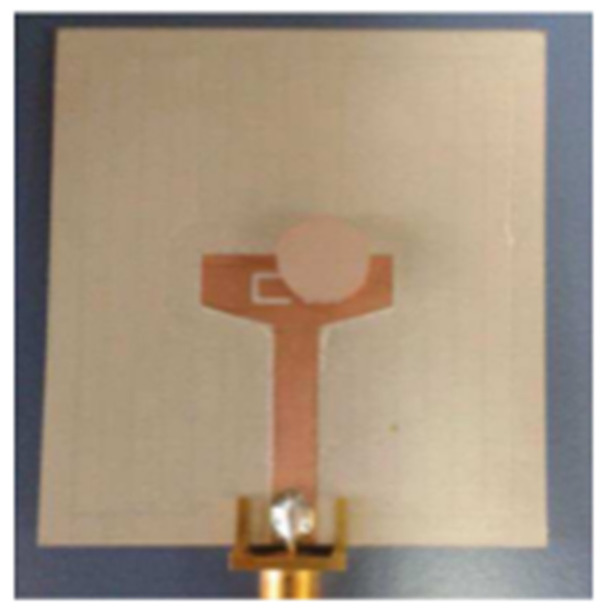	4	-	Advantage: Shows reconfiguration characteristics for microwave and WiMax applications Disadvantage: The antenna gain variations are not uniform.	Rotation DR with stepper motor	Rectangular split-ring slot	3.2–5.1
[150]	30×30	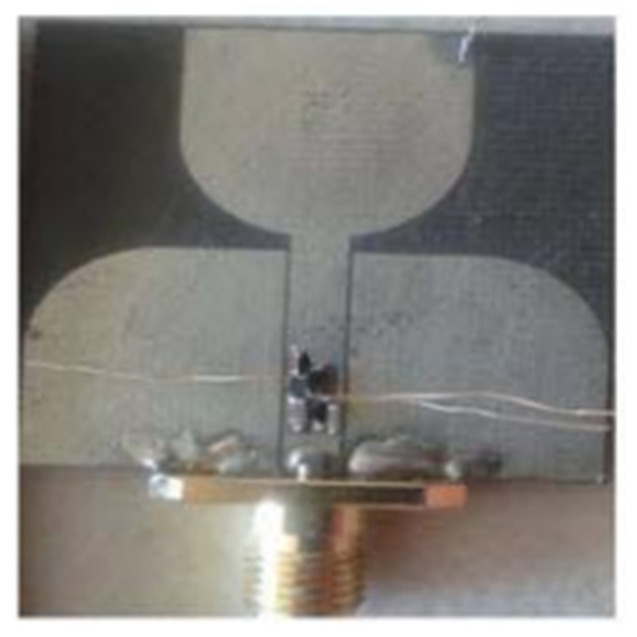	-	-	Advantage: It can mitigate UWB interference and the notch has narrowband functionality. Disadvantage: The biasing circuit utilized to obtain reconfiguration affects the gain and radiation pattern of the antenna drastically.	PIN and varactor diodes	Switchable slots	3.1–10.6
[151]	35×49.4	-	5	90	Advantage: Simple biasing structure gives suitable frequency reconfiguration and tenability Disadvantage: Tuning leads to stray capacitance.	PIN diodes and the varactor diode	CPW loaded with a switchable/tunable S-SRR along with	2.1–10.6
[152]	21×9	-	7	80	Advantage: Compact size, good gain, and radiation efficiency Disadvantage: Complex design	PIN diode	Slot Antenna with stepped slots	2.8–10.7
[153]	40×40	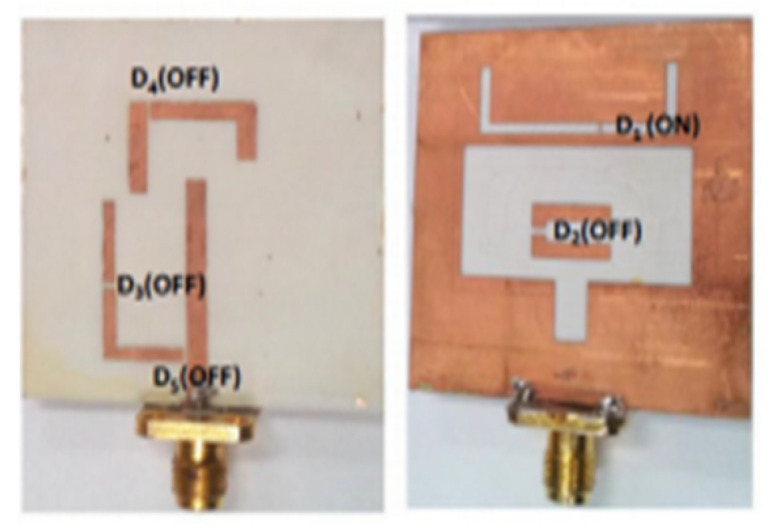	5	-	Advantage: It gives seven modes of operation Disadvantage: There is distortion in the radiation pattern at a higher frequency.	PIN diode	Three slots in the ground plane with parasitic strips on feed line and PIN diodes for switching	3.1–10.6
[154]	8×27.5	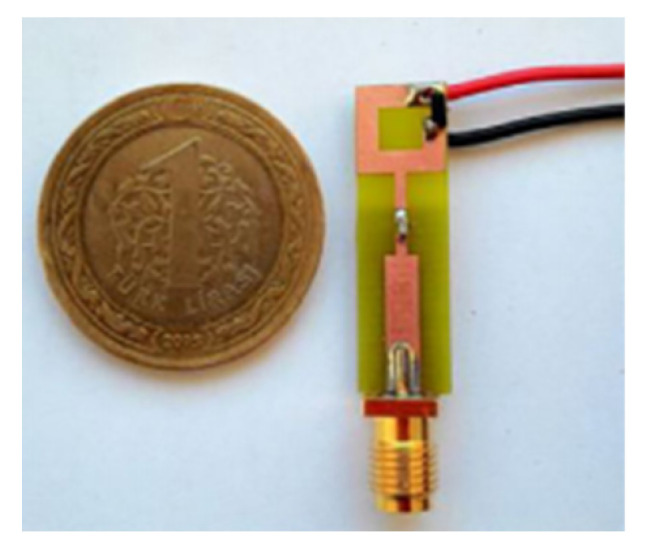	4	>70	Advantage: Compact size, stable gain and reconfigurability Disadvantage: The proposed antenna does not show stable radiations at higher operating frequencies.	PIN diode	G-shaped monopole with PIN diode	2.8–12.6
[155]	22×28	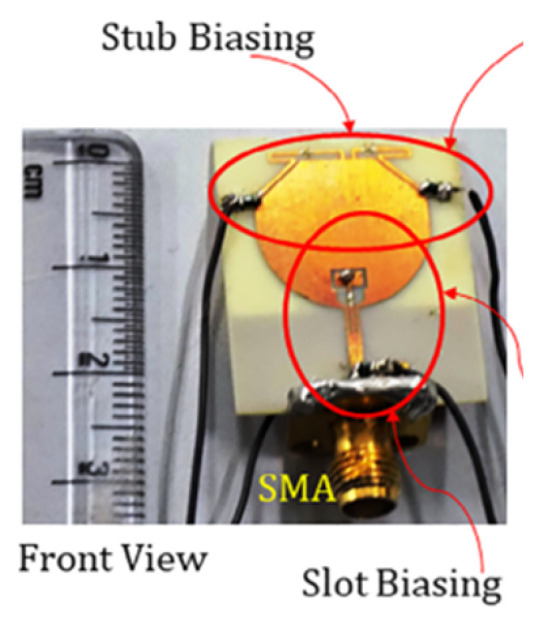	3	80–95	Advantage: Good radiation efficiency Disadvantage: The biasing circuit greatly affects the return loss, gain, and radiation pattern.	PIN diode	Monopole antenna with T-shaped slot	3–14
[156]	30×40	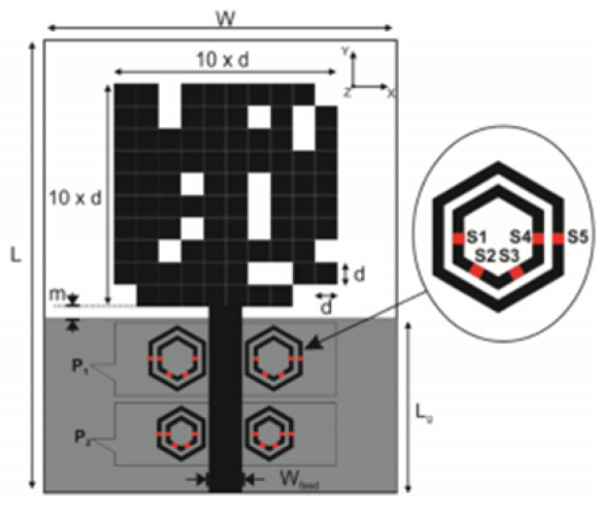	6	-	Advantage: Filters the frequency sweep of narrowband from 5.7 GHz to 8 GHz Disadvantage: Complex structure	PIN diodes	Hexagonal SRR metamaterial cells	2.8–11
[157]	34.9×31.3	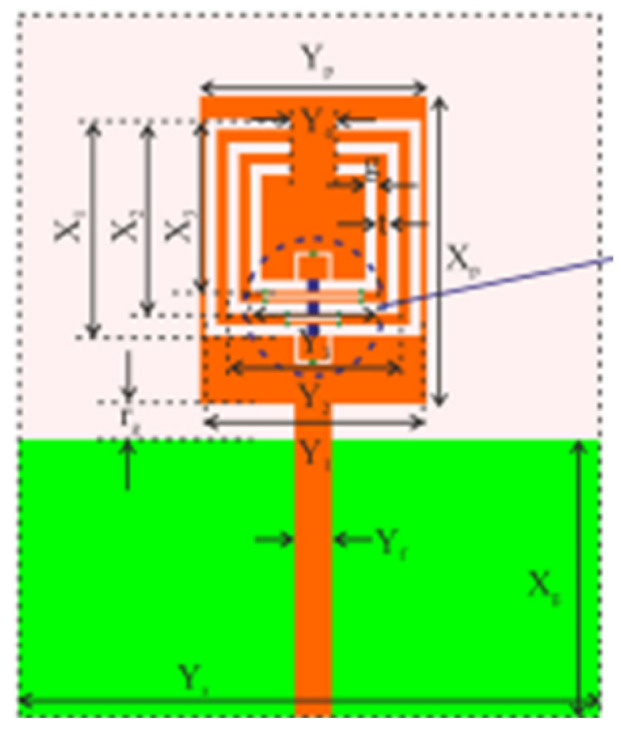	4	80–90	Advantage: Eliminates the interference of LTE WiMaz frequency range Disadvantage: Design technique affects radiation characteristics	Varactor diodes	Split-ring slot	1.9–10.5
[158]	33×21	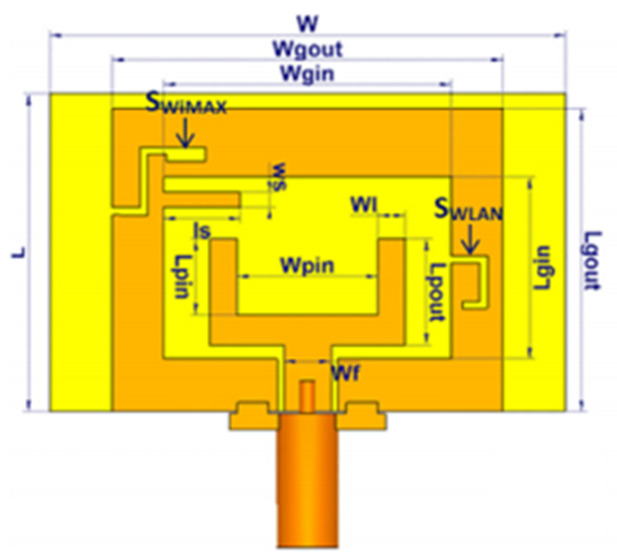	6	70	Advantage: Successfully filters the WiMax and WLAN signals from the operating band Disadvantage: Efficiency can be improved.	Use of two PIN diodes	Two open-ended slot	3–10.6

Note: - indicates not applicable.

## Data Availability

Not applicable.
